# Major adverse cardiac events with haloperidol: A meta-analysis

**DOI:** 10.1371/journal.pone.0326804

**Published:** 2025-06-25

**Authors:** Michael Cristian Garcia, Mason Anderson, Michelle Li, Mick Zewdu, Tyler Schneider, Jessyca Matos-Silva, Genevieve Ramnarine, Kassie Rong, Lawrence Mbuagbaw, Anne Holbrook

**Affiliations:** 1 Clinical Pharmacology and Toxicology Research Group, Research Institute of St Joe’s Hamilton, Hamilton, Canada; 2 Temerty Faculty of Medicine, University of Toronto, Toronto, Canada; 3 e-Health MSc Program, Faculty of Health Sciences, DeGroote School of Business, Faculty of Engineering, McMaster University, Hamilton, Canada; 4 Department of Health Research Methods, Evidence, and Impact, McMaster University, Hamilton, Canada; 5 Biostatistics Unit, Father Sean O’Sullivan Research Centre, St Joseph’s Healthcare, Hamilton, Canada; 6 Division of Clinical Pharmacology and Toxicology, Department of Medicine, McMaster University, Hamilton, Ontario, Canada; University Hospital Knappschaftskrankenhaus Bochum, GERMANY

## Abstract

**Background:**

Haloperidol is a commonly used antipsychotic drug and a frequent source of medication safety alerts because of its listing as a “known risk” QT interval-prolonging medication (QTPmed). We aimed to summarize the high-quality literature on the frequency and nature of proarrhythmic major adverse cardiac events (MACE) associated with haloperidol.

**Methods:**

We searched Medline, Embase, International Pharmaceutical Abstracts, and Cochrane Central for randomized controlled trials (RCTs) involving patients 18 years or older comparing haloperidol to placebo. The FDA-adapted MACE composite included death, non-fatal cardiac arrest, ventricular tachyarrhythmia including torsades de pointes, and seizure or syncope. Random-effects meta-analyses were performed with a treatment-arm continuity correction for single and double zero event studies.

**Results:**

84 RCTs (n = 12180, 46% female), 23.8% of trials reported mean or median ages of their participants to be older than 65 years with 37 (44.0%) involving participants with psychiatric diagnoses, and 50 (59.5%) including electrocardiograms. Median follow-up duration was 28.0 days (interquartile range [IQR]=51.0). There were 1144 events, of which 97.8% were deaths, with 22 ventricular arrhythmias and 3 seizures or syncope. There was no difference in MACE with exposure to haloperidol compared to placebo (risk ratio [RR] 0.93, 95% CI: 0.80–1.08; I^2^ = 0%). IV haloperidol was not associated with increased risk of mortality (n = 5873, RR: 0.88, 95%CI:0.72–1.08).

**Conclusions:**

We did not find that haloperidol was arrhythmogenic or increased mortality in these largely short-duration trials. Further research to clarify actual clinical outcomes related to QTPmeds is important to inform safe prescribing practices.

## Introduction

Haloperidol is a dopamine (D2) antagonist commonly used to treat psychotic disorders such as schizophrenia, delirium, behaviours of dementia, and less frequently nausea and vomiting [1–3]. Haloperidol is an older ‘first generation’ antipsychotic drug but still generates more than 1 million prescriptions annually in the USA alone [4,5]. For behaviours of dementia, although there is evidence of harm including stroke and mortality, there are few alternatives and antipsychotic drug use has been rising amongst seniors in Canada with haloperidol as the most commonly used typical antipsychotic [6,7]. Antipsychotic drugs including haloperidol are also commonly prescribed for long term care home patients in Europe [8]. Similarly haloperidol’s role in treating delirium is notable given its prevalence amongst older adults – nearly 30% of older hospitalized adults with higher rates in critical care and palliative care settings [9–16]. Haloperidol’s utilization has been increasingly replaced by newer and more expensive ‘atypical’ antipsychotics partly because of perception of improved cardiac safety.

The FDA currently recommends electrocardiogram assessments for haloperidol, particularly for intravenous (IV) formulations due to case reports of QT-prolongation (QTP), sudden death and torsades de points (TdP) [17–19]. Although this type of evidence is rated low to very low quality, haloperidol is a frequent source of medication safety alerts in most electronic medical records and other decision support systems due to QTP concerns. The contribution of haloperidol itself to life threatening arrhythmias is unclear, as many instances of TdP took place in patients with concomitant risk factors [17].

Despite the theoretical concerns related to QTP, haloperidol has had a generally favourable safety and tolerance profile. For example, in adult ICU patients with delirium (n = 1000) haloperidol treatment reduced mortality at 1-year follow-up compared to placebo (adjusted absolute risk difference −6.4% (95%CI: −12.8% to −0.2%, p = 0.045) [20]. These findings were further corroborated in a meta-analysis of ICU trials comparing haloperidol to placebo (n = 951), which did not find an effect of haloperidol on mortality (RR 1.01; 95% CI 0.33–3.06) [21]. However this analysis was limited by low certainty of evidence and fewer than ten pooled trials [22].

A network meta-analysis of randomized trials on antipsychotics for schizophrenia (n = 10,177) found no evidence of increased risk of mortality with haloperidol compared to placebo (RR:1.09, 95%CI: 0.06–19.86) [23]. There were no differences between any of the studied antipsychotics and placebo or among antipsychotics in terms of mortality. In a retrospective cohort study (n = 17,115) comparing haloperidol to other oral antipsychotics (risperidone, quetiapine, olanzapine) in older adults after major surgery, there were no statistical difference between groups for in-hospital death, or cardiac arrythmias [24]. Finally, a meta-analysis of randomized controlled trials (RCTs) exploring the mortality risk with antipsychotic use in elderly patients with dementia or delirium (N = 2387) did not find an increase in mortality with haloperidol, reporting a risk ratio of 1.25 (95%CI: 0.59–2.65) [25].

Previous reviews on the safety of haloperidol have identified a lack of research focusing on patient-important clinical endpoints [3]. Notably, there is a lack of knowledge synthesis on the cardiac safety and risk of death with haloperidol across the diverse patient populations for which it is indicated. As part of a research program to clarify the incidence of FDA-defined major adverse cardiovascular events (MACE) associated with commonly used QTPmeds, we performed a systematic review with meta-analysis of RCTs of adult patients exposed to haloperidol [26].

## Methods

We conducted and reported this systematic review in accordance with the Preferred Reporting Items for Systematic Reviews and Meta-Analyses (PRISMA) reporting guidelines [27]. The completed PRISMA checklist is included in an online supplemental file (S1 Table). This review was prospectively registered on Open Science Framework (https://osf.io/zvp9n). Ethics approval was not required for this study.

### Study identification

We searched Medline, Embase, International Pharmaceutical Abstracts, and Cochrane CENTRAL in week 4 June 2023 (S1 Fig) and repeated the search in week 2 August 2024 (S2 Fig) using search strategies drafted in collaboration with medical science librarians at the University of Toronto. Complete search strategies are available in supplementary appendices. We also completed supplemental searches on Google Scholar using key text words for placebo-controlled trials, haloperidol, safety, and adverse drug reactions. There were no date restrictions.

### Inclusion criteria

Our inclusion criteria were as follows: (i) parallel-group, factorial or crossover design RCTs of any duration; (ii) adult patients defined as age 18 years or older; (iii) at least one intervention arm where the independent effect of haloperidol was measured; (iv) a comparison arm consisting of placebo, active comparator, or no treatment. We did not exclude trials that did not report any MACE events (zero-zero event trials).

We excluded RCTs involving pediatric patients defined as age less than 18 years old, or mixed pediatric-adult populations where the adult data could not be separated. RCTs involving healthy volunteers, protocols and conference abstracts were excluded. We did not include any observational studies including health system data base analyses or post-marketing studies due to biases and confounders inherent to these study designs. Studies not written in English language were also excluded.

### Study selection

Six authors (MCG, MA,ML,MZ,JS, TS) performed title and abstract screening independently and in-duplicate in Covidence [28]. Potentially relevant publications were assessed further during full-text screening.

### Extraction of study characteristics and outcomes

Study characteristics and outcomes were extracted independently by three pairs of reviewers using forms developed in Covidence. Data extraction took place between September to December 2024. We pilot-tested and used a prospectively designed standardized data-extraction form to extract details on study characteristics, dose and frequency of study drugs, participant characteristics, cardiac exclusion criteria (e.g., history of bradycardia, QT-prolongation, TdP, ventricular arrhythmias, abnormal electrocardiograms, etc.) and MACE. Our primary outcome was the MACE composite recommended by the FDA, which included the following components: all-cause mortality, sudden cardiac death, non-fatal cardiac arrest, TdP, ventricular arrhythmia (ventricular tachycardia or ventricular fibrillation), syncope, or seizure. The FDA recognizes that attribution of cause of death in trials is challenging, and thus we used the best estimate of sudden cardiac death in randomized trials, including all-cause mortality [26]. We extracted data on whether any of our MACE or QT-prolongation were pre-specified outcomes of interest. Discrepancies and disagreements were resolved through discussion or adjudicated by a third senior author.

### Statistical analysis

We used the *meta 6.0* package in R to conduct the meta-analysis and pool trial data. The unit of analysis was individuals exposed to study medications (haloperidol or placebo) and data are reported as incident events (number of participants who developed MACE during the study period). Analysis was by intention to treat and included all participants, including dropouts, to minimize bias due to differences in dropout numbers between groups. To retain studies with zero events in both arms and in accordance with existing frameworks, we used Mantel-Haenzel odds ratios (MH ORs) with Treatment Arm Continuity Correction (TACC) along with 95% confidence intervals (CIs). When incorporating crossover trial data into the meta-analyses, only data from before the first crossover was analyzed [29]. As a post hoc analysis, we performed a meta-regression in R of the effect based on the cumulative haloperidol dose on MACE, similar to the methodology of other systematic reviews [30–32].

Statistical heterogeneity was assessed using the Cochrane’s Q statistic and I^2^ statistic [29]. Statistical significance was set at two-sided α of 0.05. In trials that had more than two intervention groups, for example two arms of haloperidol that differed by dose, we preserved randomization but collapsed the multiple intervention arms into single treatment arms [33,34]. Publication bias was assessed using funnel plots and Harbord’s test [35].

### Risk of Bias

A Risk of Bias assessment tool adapted from an expert consensus process based on systematic review of leading examples was used to examine eligible studies [36,37]. Studies were identified as high-risk of bias overall if at least 1 of 5 major domains was rated as high-risk. Domains included quality of the randomization process, deviations from intended intervention, missing data, bias due to subjectivity of outcomes, and selection of reported results. For randomization, trials were classified as low risk if they described appropriate randomization and allocation concealment procedures. For deviations from the intended intervention, trials that were double or triple blind were rated low risk if there were no important differences in co-interventions. Missing data was considered low risk if less than 10% of outcome data was missing or missing data was handled via multiple imputation. Bias in the selection of reported results was rated low if a pre-specified analysis plan, protocol, or study registration was provided [37].

### Quality of evidence

Quality of evidence generated from meta-analyses and subgroup analyses of RCTs was assessed using the Grading of Recommendations, Assessment, Development and Evaluations (GRADE) framework [38]. A GRADE summary of study findings table using GRADEPro GDT (https://gradepro.org/) was used to present GRADE ratings [39].

### Subgroup and sensitivity analyses

Pre-specified analyses for MACE were conducted for the following subgroups: > 50% vs ≤ 50% female participants; mean age < 65 vs ≥ 65 years; MACE were pre-specified outcomes of interest (yes/no); cardiac comorbidities were exclusion criteria (yes/no); duration of follow-up (days); adverse event data reported (yes/no); and risk of bias. Post-hoc analyses were conducted by route of haloperidol administration (oral, IV, intramuscular (IM) injection).

For sensitivity analyses we investigated the robustness of our effect estimates when using alternative zero-event correction approaches including the Peto statistical model approach, and the exclusion of all-cause mortality from the overall MACE composite.

## Results

**Fig 1:**
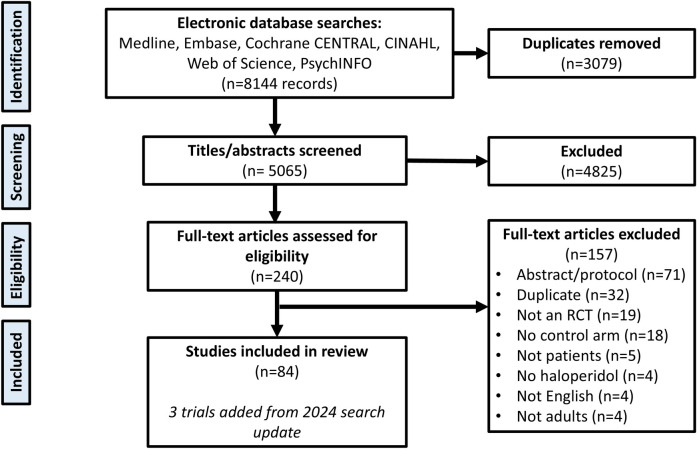
PRISMA flow diagram of the included studies in the meta-analysis. QTPmed: QT interval-prolonging medication; RCT: randomized control trial.

A total of 8144 records were retrieved from our literature searches (see complete search strategy online S1 Fig). 134 articles were retrieved in the 2024 search update, which yielded an additional three included trials. A total of 84 trials were included in this systematic review with meta-analysis (n = 12,180). Flow chart is shown in Fig 1. A list of excluded trials is provided as supplementary data (S6 Table).

### Study characteristics

Thirty-eight trials were conducted at North American sites [40–77], followed by 16 from Europe [78–93], 14 from Asia [94–107], 11 from multiple regions [108–118], three in Eastern Mediterranean [119–121], one in Oceania [122], and one in Africa [123]. Study design included 71 parallel RCTs and 13 crossover trials (detailed study characteristics are reported online S3 Table). Indications for haloperidol varied – 37 trials (n = 4171) were in psychiatry (schizophrenia, schizoaffective, bipolar disorder, obsessive compulsive disorder) [41,44–48,53–55,57,58,60,62,65,66,69,70,72–74,77,80,81,86,87,103]–[105,108,110–114,116–118], 14 (n = 2019) in surgery or perioperative patients [56,63,83,85,94–99,101,102,106,121], 10 trials (n = 1230) for those with dementia or delirium [42,49,64,75,76,78,90,91,109,122], 10 trials (n = 4142) in critically ill ICU patients [40,51,52,79,88,92,93,100,107,123], 5 trials (n = 250) for neurologic conditions (Tourette’s, migraines, headaches) [50,59,71,84,89], and 4 (n = 126) for substance use issues [43,61,82,120]. Regarding the route of haloperidol investigated, 59% of trials used oral, 32% used IV, and 8% IM injection haloperidol.

The mean trial size was 208 participants (standard deviation [SD]=249.7), with mean 46% of analyzed patients being female. 20 trials (23.8%) reported mean or median ages of their participants to be older than 65 years. The mean duration of follow-up was 61.2 days (SD:95.7), median was 28 days (IQR:51.0). Daily doses of haloperidol ranged from 1 to 40 mg daily, mean 6.9 mg. Thirty-three trials (39.3%) excluded participants with cardiovascular conditions. In terms of outcomes, 17.8% of studies listed at least one MACE in their methods as a pre-specified outcome and 58.3% of trials conducted ECG assessments.

### Risk of bias and certainty of evidence

**Fig 2:**
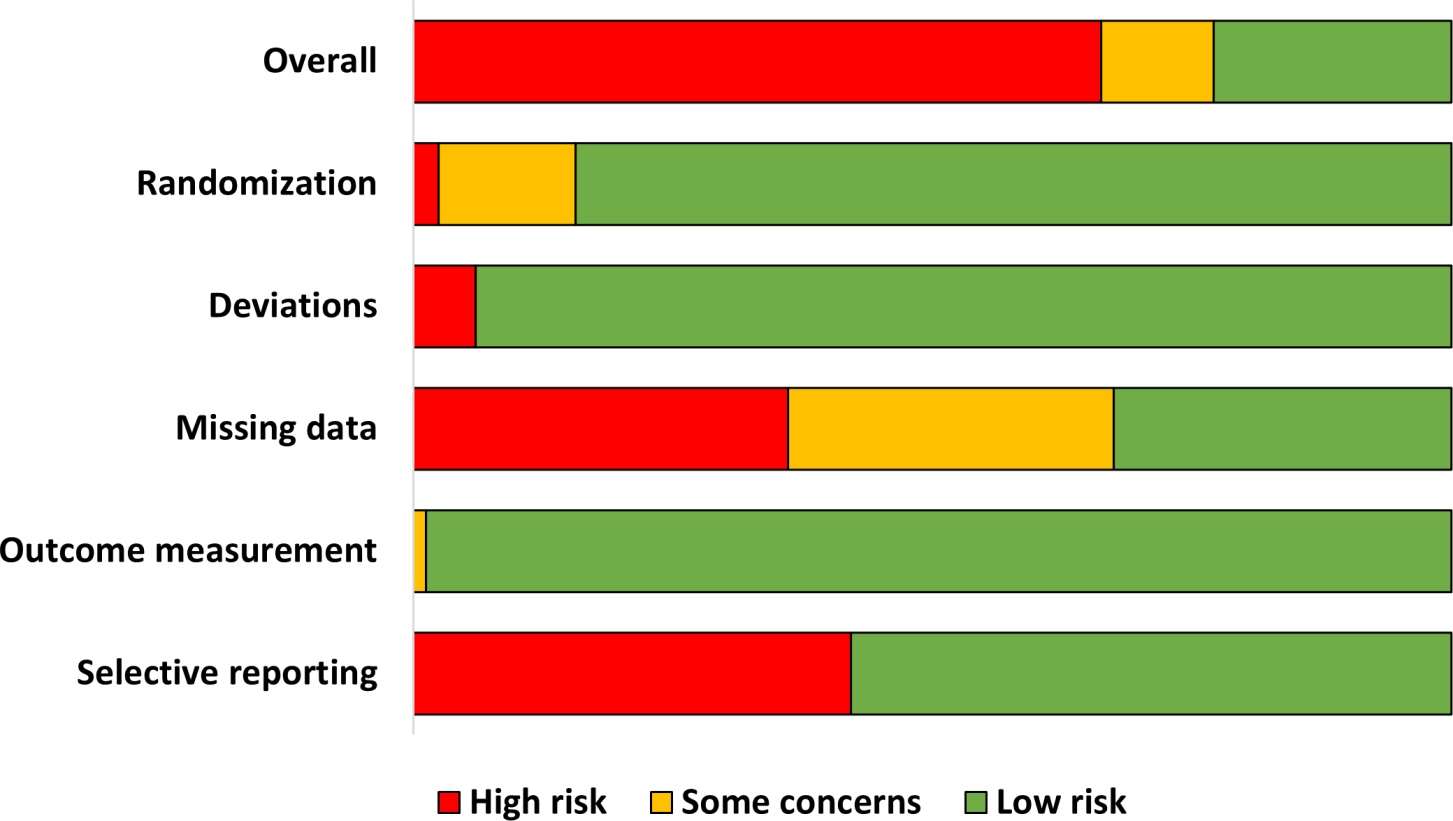
Summary of risk of bias for included trials.

Our risk of bias assessment classified 19/84 studies (23%) as low-risk of bias [40,51,52,59,73,77]–[79,83,88,92,94,95,101,107,108,114,119,122], 10% as some concerns [46–49,53,63,64,80,85], and 67% as high-risk of bias overall (Fig 2). Trials were categorized as high-risk of bias mainly due to not reporting a study protocol, registry, or pre-specified analysis plan (n = 35 RCTs) and large amounts of missing data without conducting multiple imputation to account for this missing data (n = 30 RCTs). Individual study data is available online in S4 Table.

The certainty of evidence rating was moderate for mortality and low for ventricular arrhythmias with downgrades due to risk of bias and imprecision. The absolute rate of mortality with haloperidol was 94 events per 1000 (95%CI: 74–102), compared to 101 events per 1000 without haloperidol (S2 Table). The pooled relative risk of ventricular arrhythmias was RR: 1.11, 95%CI: 0.74–1.65.

### MACE outcomes

**Fig 3:**
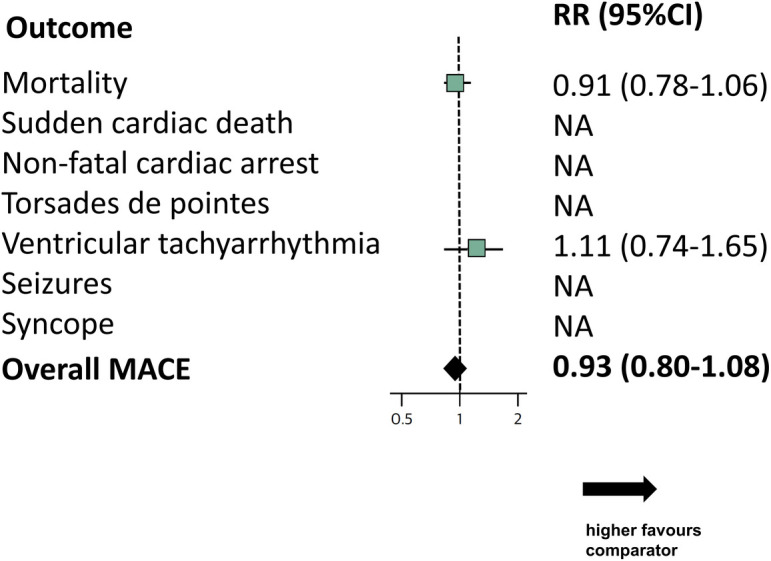
Pooled events for MACE for Haloperidol versus control. MACE: Major adverse cardiac events; RR: Risk ratio; CI: Confidence interval.

A total of 1144 MACE outcomes were detected in 17 trials [40,51,52,56,75,78,79,88,90,92,93,99,100,107,116,122,123], with the rest observing no events – 2 single-zero trials and 65 double-zero trials. Of the trials that reported MACE, over half (10/17 RCTs) were critical care trials [40,51,52,79,88,92,93,100,107,123], followed by 4 dementia and delirium trials, [75,78,90,122] two surgery or perioperative trials [56,99], and one trial in acute mania [116]. Of the 1144 MACE outcomes, 1119 (97.8%) were deaths. There were no documented events of sudden cardiac deaths or non-fatal cardiac arrests. There were 22 ventricular arrhythmias including 2 TdP (15 haloperidol, 7 placebo) [52,88,92,93,107]. There were two reported seizures (one haloperidol one placebo) and one syncope (haloperidol) [99,116]. Details of overall MACE and individual MACE composites are shown in Fig 3 and online file S4 Fig. Outcome-level data is available in online file S1 File.

Our meta-analysis shows that haloperidol was not associated with an increased risk of the MACE composite (n = 12,180, risk ratio [RR]: 0.93, 95% CI 0.80–1.08, I^2^ = 0%) or risk of mortality (RR: 0.91, 95%CI: 0.78–1.06) compared to control.

### Subgroup and sensitivity analyses

For overall MACE, mortality, or ventricular arrhythmias, subgroup analyses did not support any differences in outcomes according to mean age, female proportion, duration of follow-up, ECG assessments, cardiac exclusion criteria, prespecified MACE outcomes, absence of adverse event data, or risk of bias (details in Table 1). In a post-hoc analysis there were no subgroup differences in risk of mortality between different routes of administration (oral, IV, IM injection, p = 0.79). IV haloperidol was not associated with increased risk of mortality (RR: 0.88, 95%CI:0.72–1.08). There was no statistically significant evidence of heterogeneity within or between subgroups (Cochrane Q p-values >0.05, see S5 Table).

**Table 1:** Subgroup analyses for mortality.

Subgroup	No. of RCTs	Group; no. of events, n/N		Effect Estimate RR (95% CI)[I^2^%]
**Haloperidol**	**Control**
**Overall**	84	554/6583	565/5598	**0.91 (0.78–1.06) [0.0]**
**Patient population**				
Critical care	10	517/2271	530/1871	0.87 (0.72–1.06)[43.1]
Dementia & Delirium	10	36/615	34/615	1.09 (0.71–1.67) [0.0]
Neurologic	5	0/125	0/126	1.00 (0.18–5.60) [0.0]
Other	4	0/118	0/124	1.00 (0.14–6.96) [0.0]
Psychiatric	37	1/2266	0/1905	0.96 (0.51–1.83) [0.0]
Substance use	4	0/63	0/63	1.00 (0.15–6.80) [0.0]
Surgery/perioperative	14	0/1125	1/894	1.08 (0.39–2.99) [0.0]
**Route**				
Oral	50	47/3018	47/2732	1.01 (0.73–1.38)[0.0]
IM injection	7	0/334	0/226	1.00 (0.22–4.46)[0.0]
Intravenous route	27	507/3232	518/2641	0.88 (0.72–1.08)[0.0]
**Percent female** ^**a**^				
> 50%	30	27/1750	25/1620	1.08(0.70–1.65) [0.0]
≤50%	51	527/4760	540/3904	0.89 (0.75–1.06) [0.0]
**Age** ^**b**^				
Mean ≥65 years	20	282/2026	308/1978	0.89 (0.79–1.01) [0.0**]**
Mean <65 years	60	272/4356	257/3415	0.90 (0.70–1.16) [0.0]
**Follow-up, days (d)** ^**c**^				
≥50d	23	505/3023	513/2449	0.89 (0.73–1.09) [0.0]
<50d	58	49/3513	52/3102	0.98 (0.73–1.33) [0.0]
**ECG use at screening or baseline or follow-up**				
Yes	49	546/5279	557/4478	0.91 (0.76–1.07) [0.0]
No	35	8/1304	8/1120	0.97 (0.58–1.62) [0.0]
**Cardiac comorbidities in exclusion criteria**				
Yes	33	337/3610	328/2963	0.91 (0.74–1.12) [0.0]
No	51	217/2973	237/2635	0.88 (0.77–1.01) [0.0]
**Trial reports safety or adverse events**				
Yes	65	548/6098	558/5205	0.91 (0.77–1.08) [0.0]
No	19	6/485	7/393	0.92 (0.50–1.70) [0.0]
**MACE or component as pre-specified outcome**				
Yes	15	526/2641	540/2199	0.88 (0.73–1.06) [14.6]
No	69	28/3942	25/3399	1.06 (0.75–1.51) [0.0]
**Risk of bias**				
Low	19	510/3042	517/2468	0.90 (0.73–1.10) [0.0]
Some concerns	9	0/636	0/482	1.00 (0.27–3.71) [0.0]
High	56	44/2904	48/2647	0.96 (0.71–1.31) [0.0]

**RCTs:** Randomized controlled trials; **RR:** Risk ratio; **IM:** Intramuscular; **ECG:** Electrocardiogram; **MACE:** Major adverse cardiac events

a Missing data from three trials [54,87,97]; b missing data from four trials [47,55,70,80]; c missing data from three trials [43,82,104].

In terms of sensitivity analyses for mortality, the Peto statistical method to exclude zero-zero event trials (16 RCTs, N = 5346) produced similar effect estimates to our primary analyses, with a Peto odds ratio: 0.86, 95%CI: 0.69–1.09; I^2^ = 14.8% (Table 2). The effect estimate for overall MACE when removing mortality from the composite was RR: 1.11, 95%CI: 0.75–1.65.

**Table 2:** Sensitivity analyses for the outcome of mortality.

Statistical method	Statistical Model	No. of RCTs	Group; no. of events, n/N		Effect Estimate (95% CI)[I2%]
			Haloperidol (N = 6583)	Control (N = 5598)	
(Default) TACC	Random (MH)	84	554/6583	565/5598	RR: 0.91 (0.78–1.06) [0.0]
Peto (excluding zero-zero trials)	Random OR	16	554/ 2906	565/2440	OR: 0.86 (0.69–1.09), [14.8]
Removing all-cause mortality from MACE	Random (MH)	84	17/6583	8/5598	RR: 1.11 (0.75–1.65) [0.0]

**TACC:** Treatment arm continuity correction; **RCT:** Randomized controlled trial; **RR:** Risk Ratio; **OR:** Odds Ratio; **MH:** Mantel–Haenszel; **MACE:** Major adverse cardiac events

### Publication bias

Statistical evaluation did not suggest publication bias for overall MACE (p = 0.11) or mortality (p = 0.15). Funnel plots are provided online S3 Fig.

## Discussion

This is the first large systematic review with meta-analysis designed to understand the cardiac safety and arrhythmic potential of a commonly used medication, haloperidol, which carries warnings regarding QT interval prolongation based on low quality evidence. We found a lack of evidence that haloperidol increased proarrhythmic MACE outcomes that would be associated with serious cardiac harm – mortality, ventricular arrhythmias, seizures, or syncope. In the included 84 RCTs, non-death events were uncommon, with two reports of TdP in the haloperidol arm, 22 ventricular arrhythmias, two seizures, and one syncope. While most MACE were observed in critical care trials, we did not find an increased risk of mortality in this subgroup. This finding is consistent with previous meta-analyses on critically ill patients and adult in-patients which did not signal an increased risk of mortality [21,22,124]. Additionally, our subgroup results among patients with dementia or delirium corroborate previous meta-analyses that did not report increased risk of mortality [25]. Our effect estimates were generally robust even when removing zero-zero trials.

Our review found no statistical difference in the risk of MACE between trials that did or did not conduct electrocardiogram assessments at screening, baseline, or follow-up. Questions have been raised on the cost-effectiveness and feasibility of routine ECGs or telemetry when starting a QTPmed like haloperidol, such that compliance with ECG recommendations for antipsychotics has been low [125–129]. The Canadian Cardiovascular Society recognizes that it is not feasible to do an ECG for every patient being started on a QTPmed, rather emphasizing the importance of risk stratification and clinical judgment when considering ECG assessments [130]. UK studies have shown that performing a baseline ECG before starting haloperidol occurred in only 1.8% of patients in general practice [131,132]. Additionally an epidemiological study in Belgium (n = 222) found that only one-third of patients received a follow-up ECG while taking haloperidol despite an initial prolonged QT on baseline ECG [127]. Given more than 60 years of haloperidol use, providers appear to consider the risk of serious cardiac harm to be rare [128].

Our findings should provide some reassurance for prescribers as there was only one MACE (death in haloperidol arm) observed among 37 RCTs (n = 4,171) in psychiatric populations, and one MACE (death in placebo) among 14 RCTs (n = 2019) in surgery or perioperative populations. However, we should note that the mean ages in these groups skewed younger (37.2 years and 55.9 years, respectively).

The FDA and Health Canada report that higher than recommended doses of haloperidol are associated with higher risk of TdP [18]. A daily dose of 15 mg orally has been associated with an increase in QT interval of only 7ms, below the 10ms cut-off suggested as watchful prolongation [133]. The average daily dose in our trials at 6.9 mg was possibly low enough to minimize events. The two TdP reported in our analysis came from one critical care trial (N = 1183), however neither patient had actually received haloperidol in the 4 days immediately preceding the arrythmia [52].

Since 2007, the FDA has recommended telemetry for patients receiving IV haloperidol, and Health Canada warns against the use of IV haloperidol entirely. This was based on low quality (by modern GRADE standards) case reports and case series [134,135]. A recent 2020 systematic review including 34 clinical trials found that IV haloperidol was associated with minimal QTP or torsades de pointes until doses were very high at > 35 mg per day. Since telemetry is a scarce and costly resource, they proposed updating the FDA guidance to use in high risk patients with long QT or requiring high doses of IV haloperidol [18,134]. We found no evidence that the risk of MACE significantly differs between oral (n = 5749), IM injection (n = 559), or IV (n = 5872) routes. This may well be a true finding as prior observational studies were at risk of confounding by severity of illness (critical care patients often require parenteral forms of medication) dose and duration of treatment. To be expected, the mean daily dose (IM: 6.3 mg/d, PO: 8.5 mg/d, IV 4.1 mg/d) and duration of follow up (IM: 14 days, PO: 76 days, IV: 52 days) differed by route there was no evidence of statistical heterogeneity. As with most subgroup analyses, our findings should be interpreted as hypothesis -generating as opposed to hypothesis-testing [136]. To further validate this signal would require a dedicated and adequately sized randomized trial comparing dosage forms in hospitalized patients.

Additionally, there was no significant difference in rates of MACE between trials that performed baseline and follow-up ECGs and those that did not. While interval ECGs are recommended for patients with congenital long QT syndrome, requirements for ECG screening and monitoring for haloperidol use for the remaining majority of the population are unclear [130]. The Canadian Cardiovascular Society recently published update guidelines on managing prolonged QT intervals and recommends to ‘consider’ an ECG prior to initiation of known-risk QTPmeds if other QTP risk factors are in place [130].

The main strengths of this review are the restriction to high quality comparative studies (RCTs) to minimize the biases inherent in observational studies, moderate certainty of evidence ratings, and the comprehensive search to include 12,180 participants with diverse indications for haloperidol. Because of randomization, baseline characteristics are evenly distributed between groups, minimizing the effects of confounding due to, for example, severity of illness – an important covariate in critically ill and geriatric populations. Randomization also controls for co-intervention at baseline.

There are also several limitations, largely related to the reporting quality of the underlying trials.A potential limitation of our analysis is the variability in concomitant medication use across included trials, particularly given the limited reporting of baseline medication data. However, because the included studies were randomized, we expect that concomitant medications were balanced between treatment arms within each trial. Moreover, few studies explicitly excluded participants based on concomitant therapies such as anticoagulants, suggesting that the populations studied were broadly representative of clinical practice. While we cannot fully assess the influence of these medications due to limited reporting, we believe that their impact on the overall findings is likely minimal.

Most of the included trials did not include MACE as a pre-specified outcome in their methods and 35 of 84 trials did not mention any approach to electrocardiograms or telemetry. Moreover, 33 of 84 trials excluded patients with cardiovascular comorbidities or risk factors, which potentially limits the generalizability of our results. The mean duration of follow-up in this review was 61.2 days (SD = 95.7) and only 23 of 84 studies had a follow-up over 50 days. While this duration may capture most events in more acute inpatient or critical care settings, it may not be long enough to capture a rare incidence of MACE in outpatients since other risk factors including electrolyte disturbances, concomitant QTPmeds or metabolic inhibitors, etc. may be required to trigger events. We calculated our study to have 80% power to rule out an actual 1% MACE outcome rate in the underlying population [137].

Our certainty of evidence ratings was downgraded by issues with risk of bias, notably due to a general lack of description of the randomization procedure, proportion of missing data without multiple imputation, and absence of a pre-specified analysis plan, study protocol, or trial registry. The absence of these details is likely due to the dates of publication and the application of modern reporting standards to older trials with poorer reporting quality.

Our review adds to growing concerns regarding the validity of lower quality observational evidence used to create warnings related to ‘known’ QT prolongation by medications including haloperidol [25,138]. It aligns with our previous studies finding a lack of increased MACE with several other “known” non-cardiac QT-prolonging agents [31,32]. Our findings support a reassessment of QT-prolongation safety alerts for haloperidol in electronic medical records and pharmacy systems.

Invasive and inaccurate medication safety alerts have been shown to contribute to the administrative overload of physicians which leads to burnout and takes valuable time away from direct patient care [139–141]. The alerts are frequently overridden and may also lead to lower-quality substitute drug prescribing [142]. Since warnings regarding the non-cardiac QTPmeds are common in electronic medical records and other decision support, usually without integrating the patient’s QTc interval into the algorithms, it is important to determine whether the medications do actually cause a significant risk of MACE [143–145]. Updating clinical decision support systems based on this evidence presents challenges. Although randomized trials provide the best quality evidence, they may not include high risk patients. It is difficult to know whether haloperidol at usual doses is completely free of QT-prolongation-related cardiac harm in patients with cardiac comorbidity plus electrolyte abnormalities. A reasonable compromise would be to link the QTc-related alert to the patient’s QTc. This would be straightforward for hospital medication safety committees to institute with their digital support departments.

Research continues to evolve on how QTc is best measured and monitored, how to identify patients with congenital long QT syndrome, but the major research priority remains the development of a validated clinical prediction rule for patients who require QTPmeds to discern which factors best predict MACE [32,146,147]. Our research group is working on this.

## Conclusion

Our systematic review found that haloperidol, although listed as a “known” QTPMed, did not increase the rate of MACE including mortality. This suggests an opportunity to modify QTP-related alerts for haloperidol.

## Supporting information

S1 Table PRISMA checklist. (DOCX)

S2 Table Summary of findings table. (DOCX)

S3 Table Table of individual study characteristics. (DOCX)

S4 Table ROB table – individual studies. (DOCX)

S5 Table Statistical heterogeneity values. (DOCX)

S6 Table A list of excluded trials, with reasons. (DOCX)

S1 Fig Search Strategy – Medline May 2023. (DOCX)

S2 Fig Updated PRISMA diagram (2023–2024 update). (DOCX)

S3 Fig Funnel plot for (left) MACE, (middle) mortality, removal of double-zero trials (right). (DOCX)

S4 Fig Forest plot of haloperidol compared with control (placebo) for the incidence of any major adverse cardiac event. (PDF)

S1 File Outcome data. (XLSX)

## Abbreviations

QTP: QT-prolongation
QTPmeds: QT-interval prolonging medications
TdP: Torsade de pointes
RCTs: randomized controlled trials
MACE: major adverse cardiac events
