# Supplementary material for: Major adverse cardiac events with haloperidol: A meta-analysis
Source: PLoS One. 2025 Jun 25;20(6):e0326804. doi: 10.1371/journal.pone.0326804 (PMC12194150; doi:10.1371/journal.pone.0326804)
Supplement: S2 Table — (DOCX) [file pone.0326804.s002.docx]

**S1 Table 2. Summary of findings table for haloperidol compared to placebo**

| Outcome | Relative effect (95%CI) | Anticipated absolute effects (95%CI)^a^ | | | No. of patients  (No. of studies) | Quality of evidence (GRADE) |
| --- | --- | --- | --- | --- | --- | --- |
|  |  | Risk without Haloperidol | Risk with Haloperidol | Risk difference |  |  |
| Mortality | 0.93  (0.80-1.08) | 101 per 1,000 | 94 per, 1000  (74 fewer to 102 more) | 7 fewer per 1,000  (20 fewer to 8 more) | 12,180  (84 RCTs) | **Moderate^b^** |
| Ventricular arrhythmia | 1.11  (0.74-1.65) | 1 per 1,000 | 1 per 1,000  (1 fewer to 2 more) | 0 fewer per 1,000  (0 fewer to 1 more) | 12,180  (84 RCTs) | **Low^b,c^** |

**CI**: Confidence Interval; **GRADE**: Grading of Recommendations, Assessment, Development and Evaluations; **RR**: Risk Ratio; **RCTs**: randomized controlled trials

**GRADE Working Group quality of evidence rating**

**High quality**: We are very confident that the true effect lies close to that of the estimate of the effect

**Moderate quality**: We are moderately confident in the effect estimate; the true effect is likely to be close to the estimate of the effect, but there is a possibility that it is substantially different

**Low quality**: Our confidence in the effect estimate is limited; the true effect may be substantially different from the estimate of the effect

**Very low quality:** We have very little confidence in the effect estimate; the true effect is likely to be substantially different from the estimate of effect

^a^The risk difference (and its 95% CI) is based on the assumed risk in the comparison group and the relative effect of the intervention (and its 95% CI). An arbitrary threshold of 5% absolute risk difference is set for determining minimally important difference when assessing imprecision.

^b^Downgrade by 1 level due to risk of bias. 66% of trials were rated high risk of bias.

^c^Downgrade due to imprecision
