# Supplementary material for: Major adverse cardiac events with haloperidol: A meta-analysis
Source: PLoS One. 2025 Jun 25;20(6):e0326804. doi: 10.1371/journal.pone.0326804 (PMC12194150; doi:10.1371/journal.pone.0326804)
Supplement: S3 Table — (DOCX) [file pone.0326804.s003.docx]

**S1 Table 3:** Study characteristics

| **Study** | **Patients** | **MeanAge  % Female (F)** | **Sample size** | **Duration of FUP (days)** | **Haloperidol dose** |
| --- | --- | --- | --- | --- | --- |
| **Critical care** | | | | | |
| Van den Boogard(93) | Critically ill at high risk of delirium | Age: 66.5 F: 39% | 1796 | 90 | 1 or 2 mg IV (up to 5mg), TID |
| Mortensen 2024(80) | ICU with delirium | H:70 (10) P: 71 (13) F: 34% | 1000 | 365 | 2.5mg IV TID (up to 20mg/d) |
| Wang 2012(107) | Elderly post-operative in ICU | H:74(5.8) P:74(7.0)  F: 37% | 457 | 28 | 0.5 mg IV bolus, then continuous 0.1 mg/h for 12 hrs |
| Mart 2024(53) | ICU with delirium | H:61 (18)  P: 59 (15)  F: 43% | 376 | 365 | 2.5mg per 0.5mL or 1.25mg per 0.25mL, IV q12h (up to 20mg/d) |
| Page(89) | Mechanically ventilated in ICU | H:68(16.5) P:69(14.9)  F: 41% | 142 | 14 | 2.5mg IV, q8h |
| Smit 2023 | ICU with delirium | H: 66 (19) P: 68 (14) F: 37% | 142 | 365 | 2.5mg IV, q8h (optional titration to 5mg q8h) |
| Girard 2004(52) | Mechanically ventilated in ICU | Age:51(24)  F: 38% | 71 | 21 | 5mg in 1mg/mL solution once then q6h |
| Al-Qadheeb(41) | Critically ill with subsyndromal delirium | H:62(16.9) P:59(14.9)  F:44% | 68 | 10 | 1mg IV, q6hrs |
| Abdelgalel(123) | Non-mechanically ventilated in ICU | H: 51(8.8) P: 49(8)  F: 26% | 60 | 18 | 0.5-2mg/h continuous IV |
| Garg(100) | Critically ill with delirium | H: 57(11.5)  P: 57 (10.2)  F: 44% | 30 | 90 | up to 30mg/d PO |
| **Dementia and Delirium** | | | | | |
| Schrijver(91) | Acutely hospitalized adults | Age:84(6.3)  F: 55% | 245 | 183 | 1mg PO BID |
| DeDeyn(109) | Alzheimer’s, vascular, or mixed dementia | H:82(56-97) P:81(63-97)  F:56% | 229 | 84 | 0.25mg/mL PO BID, up to 2mg/d |
| Allain(79) | Mild/Moderate dementia with irritability/aggression | Age:80(7.6)  F:64% | 204 | 21 | 1mg PO BID, up to 6mg/d |
| Tariot(76) | Probable/Possible Alzheimer’s disease with psychosis | Age:83(6.6)  F: 73% | 193 | 70 | 0.5mg PO/d, increase by 0.5 q4d x 14d |
| Agar(122) | Palliative care with delirium | Age:75(9.8) F: 34% | 167 | 3 | 0.5mg loading IV+ 0.5mg first dose PO, then 0.5mg PO q12h (up to 4mg/d) |
| Teri(77) | Alzheimer’s with agitation | H: 75(6.9) P: 76(6.2)  F: 54% | 70 | 365 | 0.5 mg/d PO up to 3mg/d |
| Pelton(65) | Alzheimer’s with psychosis | Age:72(9.6)  F: 65% | 60 | 42 | high dose: 2 (up to 3)mg/d PO;  low dose: 0.5(up to 0.75)mg/d PO |
| Vaisanen(92) | Restless and related behavioural disturbances | Age:26(7.7)  F:33% | 30 | 42 | 5 mg BID PO, +10mg q1week up to 60 mg/d |
| Devanand(50) | Alzheimer’s with psychosis and agitation | Age: 75 (8) F: 57% | 20 | 168 | 0.5-5mg/d PO |
| Auchus(43) | Alzheimer’s with agitation | Age: 76 (7.5)  F: 67% | 12 | 42 | 3mg/d PO |
| **Psychiatric** | | | | | |
| Young (118) | Bipolar I with acute mania | H: 42 (18-76)  P: 40(18-70)  F:56% | 318 | 21 | 5mg/d up to 15mg/d PO |
| Smulevich(113) | Bipolar I disorder | H: 39(12)  P: 39(13)  F:47% | 284 | 63 | 2-12mg/d PO |
| Zimbroff(78) | Schizophrenia | Age: 39 (nr)  F:22% | 281 | 56 | 4, 8, or 16 mg/day PO |
| Daniel(49) | Schizophrenia or schizoaffective disorder | Age:42  F:39% | 273 | 5 | 6.5mg IM ; 10mg PO |
| Vieta(116) | Bipolar I with acute mania | H: 39(12.2)  P: 37(13.8)  F:41% | 260 | 21 | 8-30 mg/day PO (4, 10 or 15 mg BID), could be reduced to 4mg/d |
| Kane 2010(111) | Schizophrenia | Age: 37-40 (range)  F:32% | 238 | 42 | 4mg BID PO |
| Kane 2002(56) | Schizophrenia or schizoaffective disorder | Age: 39 (nr)  F:33% | 210 | 28 | 10mg/d PO |
| McIntyre(112) | Bipolar I disorder | Age: 43 (nr)  F:63% | 200 | 84 | 2-8mg/d PO |
| Meltzer(61) | Schizophrenia or schizoaffective disorder | H: 36(9.7)  P: 37(8.2)  F:26% | 196 | 42 | 10mg/d PO |
| Wright(117) | Schizophrenia | Age: 38 (11.6)  F:34% | 180 | 1 | 7.5mg/d IM |
| Potkin 2015(67) | Schizophrenia | Age: 41(10.1)  F: 22% | 144 | 42 | 10mg/d PO |
| Crawford(48) | Schizophrenia | H: 36(9)  P: 35(8) F:12% | 137 | 365 | 15±5mg/d PO |
| Marder(59) | Schizophrenia | Age:37(10.4)  F:12% | 132 | 56 | 20mg/d PO |
| Garcia(110) | Schizophrenia | Age:38(11.1)  F:40% | 124 | 42 | 10mg/d PO |
| Tran-Johnson(114) | Schizophrenia, schizoaffective, schizophreniform disorder | H: 41(10.2); P: 40(10.7) F:40% | 122 | 1 | 7.5mg/d IM (max 3 injections) |
| Katagiri(103) | Bipolar I disorder | H: 49(11.9); P: 43(10.9) F:55% | 117 | 21 | 2.5-10mg/d PO |
| Sachs(70) | Bipolar I disorder | H:44(20-66)  P:43(18-64) F:48% | 104 | 21 | 4-12mg/d OD PO |
| Arvanitis(42) | Schizophrenia | Age: 37 (18-64, range)  F:24% | 103 | 42 | 12mg/d TID PO |
| Breier(108) | Schizophrenia | Age: 36 (10.7)  F:43% | 85 | 1 | 7.5mg IM (max 3 injections) |
| Soloff 1993(73) | Borderline personality disorder | Age: 27(7.2)  F:76% | 70 | 35 | 4-6mg/d PO |
| Cornelius(47) | Borderline personality disorder | Age: 28(8) F: 74% | 70 | 147 | 4mg/d PO |
| Klieser(87) | Schizophrenia and major depressive disorder | Age: 43(11.6) F: 66% | 60 | 21 | 20mg/d PO |
| Soloff 1989(75) | Borderline personality disorder, shizotypal | Age:25(nr) F:75% | 56 | 25 | 2-8mg/d BID PO |
| Nishikawa 1982(105) | Schizophrenia | Age:33(8) F: 33% | 55 | Nr | 3mg BID PO |
| Nishikawa 1984(104) | Schizophrenia | H:40(9.9) H:36(8.8)  H:43(11.3)  P: 39(10)  F: 39% | 50 | 365 | 1,2,6mg/d PO |
| Soloff 1986(74) | Borderline personality disorder or schizotypal | Age:25(16-53,range) F:49% | 44 | 35 | 2mgBID (up to 12mg/d) PO |
| Ota(63) | Schizophrenia | H: 42(10.6) P:43 (8.5)  F: 20% | 44 | 90 | 10mg/d (mean) PO |
| Chouinard(46) | Schizophrenia | Age:37(10) F: 29% | 43 | 56 | 2mg/day BID increased to 20mg maintenance dose within a week PO |
| Jann(55) | Schizophrenia | H: 32(7) P:35(8.3)  F: nr% | 36 | 42 | 5mg TID up to 75mg/d max PO |
| Potkin 2001(66) | Schizophrenia | Age:35(25-47,range)  F: 0% | 27 | 39 | 10-20ug/mL PO |
| Buchsbaum(45) | Schizophrenia | Age:36(8.7) F:16% | 25 | 35 | 11mg (mean) PO |
| Serafetinides(71) | Schizophrenia | Age: 21-61(range) F:32% | 23 | 84 | 15mg concentrate |
| Li(58) | Obsessive Compulsive Disorder | Age:34(10.4) F:56% | 16 | 14 | 2mg OD PO |
| Harvey(54) | Schizophrenia | Age:48(7.5) F: 13% | 13 | 24 | 7.5mg IM once |
| Magelund(88) | Schizophrenia | Age:39(nr) F: nr% | 12 | 28 | total 1.5-15 mg/day PO |
| Browne(82) | Schizophrenia | Age:41(13.1) F:55% | 11 | 140 | 10mg/day doubled every 4wks up to 160mg/day (syrup) |
| Bateman(81) | Psychiatric patients with tardive dyskinesia | Age:55-85(range) F: 75% | 8 | 0.25 | 5mg or 10mg IV once |
| **Surgery/perioperative** | | | | | |
| Kalisvaart(86) | Elderly hip surgery | H:79(6.0)  P:80(6.3)  F:80% | 430 | 20 | 0.5mg TID PO |
| Dag(96) | Hysterectomy | H1:43(nr)  H2:38(nr) H3:42(nr)  P:47(32-76)  F:100% | 250 | 1 | 0.25 or 0.5 or 1 or 2mg once IV |
| Fukata 2017(98) | Elderly post-operative delirium | H:82(4.4)  P:81(4.3)  F:50% | 201 | 10 | 5mg/d IV |
| Joo(101) | Gynecological surgery | H:40(20-58,range)  P:40(20-60,range)  F:100% | 150 | 1 | 1 or 2mg once IV |
| Chu(95) | Hysterectomy | H:43(4.9)  P:44(4.0)  F:100% | 147 | 1 | 2mg IV once |
| Khan(57) | Noncardiac thoracic surgery | H:60(51-68)  P:62(53-69)  F:26% | 135 | 25 | 0.5mg IV TID |
| Fukata 2014(99) | Elective digestive or orthopedic surgery | H:81(0.5)  P:80(0.5)  F:47% | 121 | 7 | 2.5mg/d IV x3d |
| Parlow(64) | Elective lower limb orthopedic or urologic procedures | Age:66(8.2) F:45% | 108 | 1 | 1 or 2mg once IM |
| Wang 2008 (106) | Ambulatory laparoscopic surgery | Age:33(5.4)  F:100% | 100 | 1 | 1mg IV once |
| Ebneshahidi(97) | Elective general, gynecologic or orthopedic surgery | H:36(8)  P:33(9)  F:NR | 99 | 0.25 | 2mg once IV |
| Hollinger(84) | Elective and emergency surgery | Age:74(6.1)  F:43% | 95 | 3 | 0.005mg/kg IV once |
| Kaneko(102) | Gastrointestinal surgery | H:72(8.2)  P:73(9.3)  F:35% | 80 | 5 | 5mg/d IV |
| Aouad(94) | Gynaecological surgery | H:38(9.9)  P:38(11.2)  F:100% | 63 | 1 | 1mg IV once |
| Honermand(121) | Middle ear surgery | Age:38(15)  F:66% | 40 | 1 | 2mg IV once |
| **Neurologic** | | | | | |
| McCoy(60) | Headache in emergency department | Age:32(nr)  F:73% | 118 | 1 | 2.5mg IV once |
| Fulop(51) | Tourette’s | Age: 21(11)  F:24% | 42 | 42 | 0.15mg/kg/d (up to 10mg/d) PO |
| Honkaniemi(85) | Acute migraine | Age:36(nr)  F:85% | 40 | 28 | 5mg IV once |
| Shapiro(72) | Tourette’s | Age:21(11)  F:27% | 39 | 63 | +0.5mg q3d until response, up to10mg/d PO |
| Ransmayr(90) | Meige’s syndrome | Age:62(8.7) F:54% | 11 | 1 | 2.5mg/d IV injection |
| **Other** |  |  |  |  |  |
| Akhlaghi(119) | Emergency department adults who needed sedation | Age: 38(12) F:11% | 124 | 0.021 | 5mg IV, once |
| Tyrer(115) | Adults with intellectual disabilities | H:38(26-50) P: 43(34.5-55.5)  F: 38% | 57 | 182 | 1.25-5mg/d PO |
| Roldan(69) | Emergency department with gastroparesis | Age:46(12.5)  F:73% | 33 | 0.04 | 5mg IV once |
| Robbins(68) | Nursing home with GI disorders | Age: 72-95 (range)  F: 89% | 29 | 0.5 | 1mg IM once |
| **Substance use** | | | | | |
| Ghaderi-Bafti(120) | Opioid dependency | H: 36(7)  P: 35 (8)  F:0% | 72 | 17 | 2.5mg/d or 1.25mg/d PO |
| Berger(44) | Cocaine dependency | Age:47(14.3) F:0% | 20 | nr | 4mg PO once |
| Franken(83) | Heroin dependency | Age:36(6.4)  F:0% | 18 | nr | 2mg PO once |
| Modell(62) | Alcohol dependency | Age: 39 (22-60, range) | 16 | 14 | 0.015/0.020/0.025mg/kg, IV once |
|  |  |  |  |  |  |

**H:** Haloperidol; **P:** Placebo; **FUP:** Follow-up; **IV:** Intravenous; **PO:** Oral; **IM:** Intra-muscular; **TID**: Three times daily; **BID**: Twice daily; **ICU:** Intensive care unit; **qXhrs:** Every X hours; **mg/d:** Milligrams per day; **ug**: Microgram
Age presented as mean (SD) or median (IQR) unless otherwise specified
