# Supplementary material for: Major adverse cardiac events with haloperidol: A meta-analysis
Source: PLoS One. 2025 Jun 25;20(6):e0326804. doi: 10.1371/journal.pone.0326804 (PMC12194150; doi:10.1371/journal.pone.0326804)
Supplement: S4 Table — (DOCX) [file pone.0326804.s004.docx]

**S1 Table 4:** Risk of bias table – individual studies

| **Study** | **Randomization** | **Deviations** | **Missing data** | **Outcome measurement** | **Selective reporting** | **Overall** |
| --- | --- | --- | --- | --- | --- | --- |
| Abdelgalel 2016(123) | Low | Low | Low | Low | High | High |
| Agar 2007(122) | Low | Low | Low | Low | Low | Low |
| Akhlaghi 2019(119) | Low | Low | Low | Low | Low | Low |
| Allain 2000(79) | Low | Low | Low | Low | Low | Low |
| Al-Qadheeb 2013(41) | Low | Low | Low | Low | Low | Low |
| Mortensen 2024(80) | Low | Low | Low | Low | Low | Low |
| Aouad 2007(94) | Low | Low | Low | Low | Low | Low |
| Arvanitis 1997(42) | Low | Low | High | Low | Low | High |
| Auchus 1997(43) | Low | Low | High | Low | Low | High |
| Bateman 1979(81) | Low | Low | Some concerns | Low | Low | Some concerns |
| Berger 1996(44) | Low | Low | Some concerns | Low | High | High |
| Breier 2002(108) | Low | Low | Low | Low | Low | Low |
| Browne 1988(82) | Low | Low | High | Low | High | High |
| Buchsbaum 1992(45) | Low | Low | Some concerns | Low | High | High |
| Chouinard 1993(46) | Some concerns | Low | High | Low | High | High |
| Chu 2008(95) | Low | Low | Low | Low | Low | Low |
| Cornelius 1993(47) | Low | Low | Some concerns | Low | Low | Some concerns |
| Crawford 1997(48) | Low | Low | Some concerns | Low | Low | Some concerns |
| Dag 2020(96) | Low | Low | Some concerns | Low | High | High |
| Daniel 2007(49) | Low | Low | Some concerns | Low | Low | Some concerns |
| DeDeyn 1999(109) | Low | Low | High | Low | Low | High |
| Devanand 2011(50) | Low | Low | Some concerns | Low | Low | Some concerns |
| Ebneshahidi 2013(97) | Some concerns | Low | Some concerns | Low | High | High |
| Franken 2004(83) | Low | Low | Some concerns | Low | High | High |
| Fukata 2014(99) | High | High | Low | Some concerns | Low | High |
| Fukata 2017(98) | High | High | Low | Low | Low | High |
| Fulop 1987(51) | Some concerns | Low | Some concerns | Low | High | High |
| Garcia 2009(110) | Low | Low | High | Low | Low | High |
| Garg 2022(100) | Low | Low | Low | Low | High | High |
| Ghaderi-Bafti 2021(120) | Low | Low | High | Low | Low | High |
| Girard 2004(52) | Low | Low | Low | Low | Low | Low |
| Mart 2024(53) | Low | Low | Low | Low | Low | Low |
| Harvey 2004(54) | Low | Low | Some concerns | Low | Low | Some concerns |
| Hollinger 2015(84) | Low | Low | Low | Low | Low | Low |
| Honarmand 2012(121) | Low | Low | Some concerns | Low | High | High |
| Honkaniemi 2006(85) | Some concerns | Low | Low | Low | High | High |
| Jann 1997(55) | Some concerns | High | High | Low | High | High |
| Joo 2015(101) | Low | Low | Low | Low | Low | Low |
| Kalisvaart 2005(86) | Low | Low | Some concerns | Low | Low | Some concerns |
| Kane 2002(56) | Low | Low | High | Low | High | High |
| Kane 2010(111) | Low | Low | High | Low | Low | High |
| Kaneko 1999(102) | Some concerns | High | Some concerns | Low | High | High |
| Katagiri 2012(103) | Low | Low | High | Low | Low | High |
| Khan 2018(57) | Low | Low | High | Low | Low | High |
| Klieser 1989(87) | Some concerns | Low | High | Low | High | High |
| Li 2005(58) | Some concerns | Low | High | Low | High | High |
| Magelund 1979(88) | Low | Low | Low | Low | High | High |
| Marder 1994(59) | Low | Low | High | Low | High | High |
| McCoy 2020(60) | Low | Low | Low | Low | Low | Low |
| McIntyre 2005(112) | Low | Low | High | Low | Low | High |
| Meltzer 2004(61) | Low | Low | High | Low | Low | High |
| Modell 1993(62) | Low | Low | Some concerns | Low | High | High |
| Nishikawa 1982(105) | Low | Low | Some concerns | Low | High | High |
| Nishikawa 1984(104) | Low | Low | Some concerns | Low | High | High |
| Ota 1973(63) | Low | Low | High | Low | Low | High |
| Page 2010(89) | Low | Low | Low | Low | Low | Low |
| Parlow 2004(64) | Low | Low | Some concerns | Low | Low | Some concerns |
| Pelton 2003(65) | Low | Low | Some concerns | Low | Low | Some concerns |
| Potkin 2001(66) | Low | Low | Some concerns | Low | High | High |
| Potkin 2015(67) | Low | Low | High | Low | High | High |
| Ransmayr 1988(90) | Some concerns | High | High | Low | High | High |
| Robbins 1975(68) | Low | Low | High | Low | High | High |
| Roldan 2015(69) | Low | Low | High | Low | Low | High |
| Sachs 2002(70) | Low | Low | High | Low | Low | High |
| Schrijver 2018(91) | Low | Low | High | Low | Low | High |
| Serafetinides 1972(71) | Low | Low | High | Low | High | High |
| Shapiro 1989(72) | Low | Low | Some concerns | Low | High | High |
| Smit 2023 | Low | Low | High | Low | Low | High |
| Smulevich 2005(113) | Low | Low | Some concerns | Low | High | High |
| Soloff 1986(74) | Low | Low | Low | Low | Low | Low |
| Soloff 1989(75) | Some concerns | Low | Some concerns | Low | High | High |
| Soloff 1993(73) | Low | Low | Some concerns | Low | High | High |
| Tariot 2006(76) | Low | Low | High | Low | High | High |
| Teri 2000(77) | Low | Low | High | Low | Low | High |
| Tran-Johnson 2007(114) | Low | Low | Low | Low | Low | Low |
| Tyrer 2009(115) | Low | Low | High | Low | Low | High |
| Vaisanen 1981(92) | Low | Low | Low | Low | High | High |
| van den Boogaard(93) | Low | Low | Low | Low | Low | Low |
| Vieta 2010(116) | Some concerns | Low | High | Low | Low | High |
| Wang 2008(106) | Low | Low | Low | Low | High | High |
| Wang 2012(107) | Low | Low | Low | Low | Low | Low |
| Wright 2003(117) | Low | Low | Some concerns | Low | High | High |
| Young 2009(118) | Low | Low | High | Low | Low | High |
| Zimbroff 1997(78) | Low | Low | Low | Low | Low | Low |
