# Supplementary material for: Major adverse cardiac events with haloperidol: A meta-analysis
Source: PLoS One. 2025 Jun 25;20(6):e0326804. doi: 10.1371/journal.pone.0326804 (PMC12194150; doi:10.1371/journal.pone.0326804)
Supplement: S5 Table — (DOCX) [file pone.0326804.s005.docx]

**S1 Table 5:** Statistical heterogeneity values (mortality) for subgroup and sensitivity analyses

| **Subgroup** | **Cochrane Q (p-value)** | **I^2^ % (95% CI)** |
| --- | --- | --- |
| **Overall** | 19.05 (p=1.00) | 0.0% (0.0-26.4) |
| **Patient population** | 1.03 (p=0.98) |  |
| Critical care | 15.83 | 43.1% |
| Dementia & Delirium | 1.22 | 0.0% |
| Neurologic | 0.00 | 0.0% |
| Other | 0.00 | 0.0% |
| Psychiatric | 0.23 | 0.0% |
| Substance use | 0.00 | 0.0% |
| Surgery/perioperative | 0.41 | 0.0% |
| **Route** | 0.48 (p=0.79) |  |
| Oral | 2.16 | 0.0% |
| IM injection | 0.00 | 0.0% |
| Intravenous route | 16.11 | 0.0% |
| **Percent female^a^** | 0.68 (p=0.71) |  |
| > 50% | 1.26 | 0.0% |
| ≤50% | 16.88 | 0.0% |
| **Age^b^** | 0.02 (p=0.99) |  |
| Mean ≥65 years | 4.11 | 0.0% |
| Mean <65 years | 14.84 | 0.0% |
| **Follow-up, days (d)^c^** | 0.28 (p=0.87) |  |
| ≥50d | 14.58 | 0.0% |
| <50d | 3.87 | 0.0% |
| **ECG use** | 0.07 (p=0.79) |  |
| Yes | 18.43 | 0.0% |
| No | 0.47 | 0.0% |
| **Cardiac comorbidities in exclusion criteria** | 0.07 (p=0.79) |  |
| Yes | 18.05 | 0.0% |
| No | 1.00 | 0.0% |
| **Trial reports safety or adverse events** | 0.00 (p=0.98) |  |
| Yes | 18.97 | 0.0% |
| No | 0.06 | 0.0% |
| **MACE pre-specified** | 0.86 (p=0.35) |  |
| Yes | 16.40 | 14.6% |
| No | 1.50 | 0.0% |
| **Risk of bias** | 0.14 (p=0.93) |  |
| Low | 16.46 | 0.0% |
| Some concerns | 0.00 | 0.0% |
| High | 2.23 | 0.0% |
| **Statistical method** |  |  |
| Peto | 17.61 (p=0.28) | 14.8% (0.0-51.9) |
| Removing all-cause mortality from MACE | 6.19 (p=1.00) | 0.0% (0.0-26.4) |
