# Supplementary material for: Major adverse cardiac events with haloperidol: A meta-analysis
Source: PLoS One. 2025 Jun 25;20(6):e0326804. doi: 10.1371/journal.pone.0326804 (PMC12194150; doi:10.1371/journal.pone.0326804)
Supplement: S6 Table — (DOCX) [file pone.0326804.s006.docx]

**S2 Table 1 – Studies excluded from review, with exclusion reason**

| **Authors** | **Year**  **published** | **Title** | **Exclusion reason** |
| --- | --- | --- | --- |
| Agar, M; Lawlor, P; Quinn, S; Draper, B; Caplan, G; Rowett, D; Sanderson, C; Hardy, J; Le, B; Eckermann, S; et al. | 2016 | Phase III randomized double-blind controlled trial of oral risperidone, haloperidol or placebo with rescue subcutaneous midazolam for delirium management in palliative care | Duplicate |
| Agar, MR; Lawlor, P; Draper, B; Caplan, G; Hill, M; Rowett, D; Sanderson, C; Quinn, S; Eckermann, S; McCaffrey, N; et al. | 2014 | Randomised control trial of oral risperidone, oral haloperidol, and oral placebo with rescue subcutaneous midazolam in the management of delirium in palliative care inpatients | Duplicate |
| Agar, MR; Lawlor, PG; Quinn, S; Draper, B; Caplan, GA; Rowett, D; Sanderson, C; Hardy, J; Le, B; Eckermann, S; et al. | 2017 | Efficacy of Oral Risperidone, Haloperidol, or Placebo for Symptoms of Delirium Among Patients in Palliative Care: a Randomized Clinical Trial | Duplicate |
| Ahc, M | 2019 | Antipsychotics do not shorten the duration of ICU delirium | No full-text |
| Al-Qadheeb, NS; Skrobik, Y; Schumaker, G; Pacheco, MN; Roberts, RJ; Ruthazer, RR; Devlin, JW | 2016 | Preventing ICU Subsyndromal Delirium Conversion to Delirium With Low-Dose IV Haloperidol: a Double-Blind, Placebo-Controlled Pilot Study | Duplicate |
| Aman, MG; Teehan, CJ; White, AJ; Turbott, SH; Vaithianathan, C | 1989 | Haloperidol treatment with chronically medicated residents: dose effects on clinical behavior and reinforcement contingencies | No full-text |
| Andrezina, R; Josiassen, RC; Marcus, RN; Oren, DA; Manos, G; Stock, E; Carson, WH; Iwamoto, T | 2006 | Intramuscular aripiprazole for the treatment of acute agitation in patients with schizophrenia or schizoaffective disorder: a double-blind, placebo-controlled comparison with intramuscular haloperidol | Duplicate |
| Arvanitis, LA; Miller, BG | 1996 | Quetiapine, an Atypical Antipsychotic - Results From a Multiple Fixed Dose, Placebo-Controlled Study | No full-text |
| AstraZeneca, | 2006 | An International, Multicenter, Double-blind, Randomized, Placebo-controlled Study of the Safety and Efficacy of Seroquel (Quetiapine Fumarate) and Haloperidol as Monotherapy in the Treatment of Acute Mania | No full-text |
| Ayesa-Arriola, R; RodrÃ­guez-SÃ¡nchez, JM; PÃ©rez-Iglesias, R; Roiz-SantiÃ¡Ã±ez, R; MartÃ­nez-GarcÃ­a, O; SÃ¡nchez-Moreno, J; TabarÃ©s-Seisdedos, R; VÃ¡zquez-Barquero, JL; Crespo-Facorro, B | 2013 | Long-term (3-year) neurocognitive effectiveness of antipsychotic medications in first-episode non-affective psychosis: a randomized comparison of haloperidol, olanzapine, and risperidone | No placebo arm |
| Ballard, C; Hanney, ML; Theodoulou, M; Douglas, S; McShane, R; Kossakowski, K; Gill, R; Juszczak, E; Yu, LM; Jacoby, R | 2009 | The dementia antipsychotic withdrawal trial (DART-AD): long-term follow-up of a randomised placebo-controlled trial | No haloperidol arm |
| Barbier Ph. J.; Dorf G.; Gordin J.; Krainik F.; Neveu D.; Parlier H.; Richard Ph.; Vitaux J.; Fraitag B. | 1989 | Effect of the combination of buzepide methiodide and haloperidol in the treatment of the irritable bowel syndrome. A double-blind randomized placebo-controlled trial | No full-text |
| Barton, MD; Libonati, M; Cohen, PJ | 1975 | The use of haloperidol for treatment of postoperative nausea and vomiting--a double-blind placebo-controlled trial | Pediatric population |
| Battaglia, J; Lindborg, SR; Alaka, K; Meehan, K; Wright, P | 2003 | Calming versus sedative effects of intramuscular olanzapine in agitated patients | Not an RCT |
| Beasley, CM; Dellva, MA; Tamura, RN; Morgenstern, H; Glazer, WM; Ferguson, K | 1999 | A randomised double-blind comparison of the incidence of tardive dyskinesia in patients with schizophrenia during long-term treatment with olanzapine or haloperidol | Not an RCT |
| Beasley, CM; Tollefson, G; Tran, P; Satterlee, W; Sanger, T; Hamilton, S | 1996 | Olanzapine versus placebo and haloperidol: acute phase results of the North American double-blind olanzapine trial | Duplicate |
| Bechelli, LP; Ruffino-Netto, A; Hetem, G | 1983 | A double-blind controlled trial of pipotiazine, haloperidol and placebo in recently-hospitalized acute schizophrenic patients | No full-text |
| Bechelli, LPC; Iecco, MC; Acioli, A; Pontes, MC | 1985 | A double-blind trial of haloperidol decanoate and pipothiazine palmitate in the maintenance treatment of schizophrenics in a public out-patient clinic | No placebo arm |
| Borger van der Burg, BLS | 2005 | Randomised Double-Blind Placebo-Controlled Study of Post-Operative Haloperidol Versus Placebo for Prevention of Post-Operative Delirium After Acute Hip Surgery | No full-text |
| Borger vand der Burg, B | 2006 | Post-operative Haloperidol versus Placebo for prevention of post-operative delirium after acute hip surgery | No full-text |
| Borison, RL; Arvanitist, LA; Miller, BG | 1996 | A comparison of five fixed doses 'seroquel' (ici 204,636) with haloperidol and placebo in patients with schizophrenia conference proceeding | No full-text |
| Borison, RL; Sinha, D; Haverstock, S; McLarnon, MC; Diamond, BI | 1989 | Efficacy and safety of tiospirone vs. haloperidol and thioridazine in a double-blind, placebo-controlled trial | No full-text |
| Bowden, CL; Myers, JE; Grossman, F; Xie, Y | 2004 | Risperidone in combination with mood stabilizers: a 10-week continuation phase study in bipolar I disorder | No haloperidol arm |
| Brauer, LH; Cramblett, MJ; Paxton, DA; Rose, JE | 2001 | Haloperidol reduces smoking of both nicotine-containing and denicotinized cigarettes | Healthy volunteers |
| Breier, A; Wright, P; Birkett, M; Meehan, K; David, S; Brook, S | 2000 | A double-blind dose response study comparing intramuscular olanzapine, haloperidol and placebo in acutely agitated schizophrenic patients | Duplicate |
| Bristol-Myers, Squibb | 2003 | A multicenter, randomized, double-blind study of aripiprazole and haloperidol in the maintained response to treatment for an acute manic episode | No full-text |
| Cantillon, M; Goldstein, JM | 1998 | Efficacy of Quetiapine Fumarate in Affective Symptoms of Schizophrenia | No full-text |
| Carson, WH; Ali, M; Dunbar, G; Ingenito, G; Saha, AR | 2001 | A double-blind, placebo-controlled trial of aripiprazole and haloperidol | No full-text |
| Carson, WH; Ali, M; Saha, AR; Dunbar, GC; Ingenito, G | 2000 | A double-blind, placebo-controlled trial of aripiprazole and haloperidol in patients with schizophrenia or schizoaffective disorder | No full-text |
| Cazorla P.; Zhao J.; Szegedi A. | 2012 | Incidence, onset, and duration of treatmentemergent somnolence with asenapine in patients with schizophrenia or bipolar disorder | No full-text |
| Chaparro, LE; Gallo, T; Gonzalez, NJ; Rivera, MF; Peng, PW | 2010 | Effectiveness of combined haloperidol and dexamethasone versus dexamethasone only for postoperative nausea and vomiting in high-risk day surgery patients: a randomized blinded trial | No placebo arm |
| ChiCTR-TRC-09000379, | 2009 | Effect of haloperidol on early postoperative delirium and late postoperative outcomes in elderly patients after non-cardiac surgery: a multi-central, randomized controlled trial | No full-text |
| Chouinard, G | 1995 | Effects of risperidone in tardive dyskinesia: an analysis of the Canadian multicenter risperidone study | Not an RCT |
| Cornelius, JR; Soloff, PH; George, A; Ulrich, RF; Perel, JM | 1993 | Haloperidol vs. phenelzine in continuation therapy of borderline disorder | Duplicate |
| Crawford, AM; Beasley, CM; Tollefson, GD | 1996 | Olanzapine - Impact of An Atypical Antipsychotic Candidate on Prolactin Release | No full-text |
| Crawford, AMK; Beasley, CM; Tollefson, GD | 1997 | The acute and long-term effect of olanzapine compared with placebo and haloperidol on serum prolactin concentration | Duplicate |
| Cucchiaro, J; Nann-Vernotica, E; Lasser, R; Matkovits-Gupta, T; Fairweather, D; Young, F; El-Bizri, H; Klonowski, E; Narurkar, M; Gharabawi, G | 2001 | A randomized, double-blind, multicenter phase iii study of iloperidone versus haloperidol and placebo in patients with schizophrenia or schizoaffective disorder | No full-text |
| Czobor P.; Volavka J. | 1992 | Level of haloperidol in plasma is related to electroencephalographic findings in patients who improve | Not an RCT |
| David, SR; Meehan, K; Birkett, MA; Wright, P; Ferchland, I; Alaka, K; Brook, S | 2001 | Intramuscular olanzapine versus intramuscular haloperidol and intramuscular placebo: an international double-blind study in acutely agitated patients with schizophrenia | No placebo arm |
| Devanand D.P.; Pelton G.H. | 2011 | A 6-month, randomized, double-blind, placebo-controlled pilot discontinuation trial following response to haloperidol treatment of psychosis and agitation in Alzheimer's disease | Duplicate |
| Devanand, DP; Marder, K; Michaels, K; Sackeim, HA; Bell, K; Sullivan, M; Cooper, T; Mayeux, R | 1997 | A randomized, placebo-controlled, dose-comparison trial of haloperidol treatment for the behavioral complications of Alzheimer's disease | Duplicate |
| Devanand, DP; Marder, K; Michaels, KS; Sackeim, HA; Bell, K; Sullivan, MA; Cooper, TB; Pelton, GH; Mayeux, R | 1998 | A randomized, placebo-controlled dose-comparison trial of haloperidol for psychosis and disruptive behaviors in Alzheimer's disease | Duplicate |
| Eklund, K; Forsman, A | 1991 | Minimal effective dose and relapse--double-blind trial: haloperidol decanoate vs. placebo | No full-text |
| Eli, Lilly | 2006 | Placebo- and haloperidol-controlled double-blind trial of olanzapine in patients with manic or mixed episode of Bipolar I Disorder | No full-text |
| Ely, EW | 2004 | Delirium in the ICU: a prospective, randomized, trial of placebo vs haloperidol vs ziprasidone | No full-text |
| Eskander, FSL; Hakim, KYK; Ibrahim, DA; El-Mallah, WS | 2021 | Incidence Of Post-Operative Delirium In Geriatric Patients Undergoing Hip Replacement Surgery Using Pre-Operative Haloperidol | No full-text |
| EUCTR2008-007384-17-SE, | 2009 | A prospective, multi-centre, randomised, double-blind, placebo-controlled comparison of intravenous dexmedetomidine with haloperidol in treatment of hyperactive delirium after heart surgery. - Dex-in-delir | No full-text |
| Fakra, E; Salgado-Pineda, P; Besnier, N; Azorin, JM; Blin, O | 2009 | Risperidone versus haloperidol for facial affect recognition in schizophrenia: findings from a randomised study | No placebo arm |
| Feng, PH; Chu, KS; Lu, IC; Shieh, JP; Tzeng, JI; Ho, ST; Wang, JJ; Chu, CC | 2009 | Haloperidol plus ondansetron prevents postoperative nausea and vomiting in patients undergoing laparoscopic cholecystectomy | No placebo arm |
| Garver D.L.; Holcomb J.A.; Christensen J.D. | 2005 | Cerebral cortical gray expansion associated with two second-generation antipsychotics | No placebo arm |
| Giannini, AJ; Loiselle, RH; DiMarzio, LR; Giannini, MC | 1987 | Augmentation of haloperidol by ascorbic acid in phencyclidine intoxication | No full-text |
| Girard, TD; Pandharipande, PP; Carson, SS; Schmidt, GA; Wright, PE; Canonico, AE; Pun, BT; Thompson, JL; Shintani, AK; Meltzer, HY; et al. | 2010 | Feasibility, efficacy, and safety of antipsychotics for intensive care unit delirium: the MIND randomized, placebo-controlled trial | Duplicate |
| Glazer, WM; Hafez, HM; Benarroche, CL | 1985 | Molindone and haloperidol in tardive dyskinesia | No full-text |
| Goode, DJ; Manning, AA | 1983 | Comparison of bupropion alone and with haloperidol in schizo-affective disorder, depressed type | No placebo arm |
| Hamilton, SH; Revicki, DA; Genduso, LA; Beasley, CM | 1998 | Olanzapine versus placebo and haloperidol: quality of life and efficacy results of the North American double-blind trial | Duplicate |
| Hirst Jeremy, M; Vaughan Christina, L; Irwin Scott, A | 2017 | Delirium: use antipsychotics when appropriate and appropriately...Agar MR, Lawlor PG, Quinn S, et al.: efficacy of oral ris- peridone, haloperidol, or placebo for symptoms of delirium among patients in palliative care: a randomized clinical trial. JAMA I | Not an RCT |
| Hollinger, A; RÃ¼st, CA; Riegger, H; Gysi, B; Tran, F; BrÃ¼gger, J; Huber, J; Toft, K; Surbeck, M; Schmid, HR; et al. | 2021 | Ketamine vs. haloperidol for prevention of cognitive dysfunction and postoperative delirium: a phase IV multicentre randomised placebo-controlled double-blind clinical trial | Duplicate |
| Hong, WW; Arvanitis, LA; Miller, BG | 1996 | Quetiapine Does Not Differ From Placebo in the Incidence of Extrapyramidal Syndrome or Effect on Plasma Prolactin | No full-text |
| Hu, H; Deng, W; Yang, H; Liu, Y | 2006 | Olanzapine and haloperidol for senile delirium: a randomized controlled observation | No full-text |
| Hurst, BC; Link, C | 1996 | A Comparison of the Tolerance Profile of Seroquel(tm) with Typical Neuroleptics and Placebo | No full-text |
| Janssen Pharmaceutica NV, B | 2005 | Risperidone in the treatment of behavioral disturbances in demented patients: an international, multicenter, placebo-controlled, double-blind, parallel-group trial using haloperidol as internal reference | No full-text |
| Janssen Pharmaceutica, NV | 2016 | Risperidone in the Treatment of Behavioral Disturbances in Demented Patients: an International, Multicenter, Placebo-Controlled, Double-Blind, Parallel-Group Trial Using Haloperidol as Internal Reference | No full-text |
| Kalisvaart, K; Bogaards, M; de Jonghe, J; Egberts, E; Eikelenboom, P; Van Gool, P | 2002 | A placebo-controlled post-operative delirium study of haloperidol-prophylaxis in elderly hip-surgery patients | No full-text |
| Kalisvaart, KJ; De Jonghe, J; Bogaards, M; Egberts, T; Eikelenboom, P; Van Gool, P | 2003 | A placebo-controlled study of haloperidol prophylaxis for post-operative delirium in elderly hip-surgery patients | No full-text |
| Kane John, M; Ingenito, Gary; Ali, Mirza | 2002 | Efficacy of aripiprazole in psychotic disorders: comparison with haloperidol and placebo | No full-text |
| Kapur, S; Arenovich, T; Agid, O; Zipursky, R; Lindborg, S; Jones, B | 2005 | Evidence for onset of antipsychotic effects within the first 24 hours of treatment | Duplicate |
| Katagiri H.; Takita Y.; Tohen M.; Takahashi M. | 2010 | Efficacy and safety of olanzapine in the treatment of Japanese patients with a bipolar manic or mixed episode.- A multicenter, randomized, double-blind, parallel, placebo- and haloperidol-controlled study | No full-text |
| Keefe R.S.E.; Seidman L.J.; Christensen B.K.; Hamer R.M.; Sharma T.; Sitskoorn M.M.; Lewine R.R.J.; Yurgelun-Todd D.A.; Gur R.C.; Tohen M.; Tollefson G.D.; Sanger T.M.; Lieberman J.A. | 2009 | Comparative effect of atypical and conventional antipsychotic drugs on neurocognition in first-episode psychosis: A randomized, double-blind trial of olanzapine versus low doses of haloperidol (American Journal of Psychiatry (2004) 161, (985-995)) | No placebo arm |
| Khan, BA; Perkins, AJ; Campbell, NL; Gao, S; Farber, MO; Wang, S; Khan, SH; Zarzaur, BL; Boustani, MA | 2019 | Pharmacological Management of Delirium in the Intensive Care Unit: a Randomized Pragmatic Clinical Trial | No haloperidol arm |
| King, R; Fay, G; Wheildon, H; Sloman, L; Konstantareas, M; Remington, G | 2002 | Re: clomipramine vs. Haloperidol in the treatment of autistic disorder: a double-blind, placebo, crossover study | Not an RCT |
| Klieser E.; Lehmann E. | 1989 | Experimental examination of trazodone | Duplicate |
| Larson, GL; Mack, RJ; Zborowski, J; Morris, DD; Sebree, TB; Wallin, BA | 1996 | Three Doses Each of Sertindole and Haloperidol in Schizophrenics | No full-text |
| Lee, DS; Kim, MJ; Kim, SG | 1998 | Effect of Haloperidol on Alcohol Craving in Patients with Alcohol Dependence | Duplicate |
| Lee, J; Holbrook, A | 2017 | 2017 - In patients receiving palliative care, risperidone or haloperidol increased delirium symptoms vs placebo | No full-text |
| Lee, J; Holbrook, A; Agar, M | 2017 | In patients receiving palliative care, risperidone or haloperidol increased delirium symptoms vs placebo | Not an RCT |
| Lejoyeux, M; Gorwood, P; Stalla-Bourdillon, A; Ades, J | 1993 | French translation and validation of the Simpson and Angus extrapyramidal side effects rating scale. TRADUCTION ET UTILISATION DE L'ECHELLE DE SIMPSON ET ANGUS DE SYMPTOMES EXTRA-PYRAMIDAUX | No full-text |
| Lemmer, W; Klieser, E; Klimke, A | 1993 | Experimental comparison of the efficacy of the dopamine autoreceptor agonist pramipexole versus haloperidol and placebo in acute schizophrenics | No full-text |
| Leucht, S; Zhao, J | 2013 | Early improvement as a predictor of treatment response and remission in patients with acute schizophrenia: effects of asenapine | Not an RCT |
| Lieberman, JA; Tollefson, G; Tohen, M; Green, AI; Gur, RE; Kahn, R; McEvoy, J; Perkins, D; Sharma, T; Zipursky, R; et al. | 2003 | Comparative efficacy and safety of atypical and conventional antipsychotic drugs in first-episode psychosis: a randomized, double-blind trial of olanzapine versus haloperidol | No placebo arm |
| Luijten M.; Veltman D.J.; Hester R.; Smits M.; Pepplinkhuizen L.; Franken I.H.A. | 2012 | Brain activation associated with attentional bias in smokers is modulated by a dopamine antagonist | Healthy volunteers |
| Malaspina, D; Dalack, G; Leitman, D; Corcoran, C; Amador, XF; Yale, S; Glassman, A; Gorman, JM | 2002 | Low heart rate variability is not caused by typical neuroleptics in schizophrenia patients | No haloperidol arm |
| Masoumi, K; Delirrooyfard, A; Salehzadeh, M | 2019 | Comparison of the analgesic effects of haloperidol with or without morphine in patients with acute renal colic: a randomized double-blind clinical trial study | No placebo arm |
| Matzner G.; Uriarte V.; Ruether E. | 1978 | Extrapyramidal side-effects with 1/day dosage of haloperidol | No full-text |
| McCoy, JJ; Petersen, JB; Aldy, KN; Arnall, EJ | 2017 | Treatment of headache in the emergency department: haloperidol in the acute setting | Pediatric population |
| McDougle, CJ; Goodman, WK; Leckman, JF; Lee, NC; Heninger, GR; Price, LH | 1994 | Haloperidol addition in fluvoxamine-refractory obsessive-compulsive disorder. A double-blind, placebo-controlled study in patients with and without tics | No placebo arm |
| McEvoy J.P. | 1994 | Efficacy of risperidone on positive features of schizophrenia | No full-text |
| Meltzer H.; Cohen M.; Snow-Adami L.; Mackle M.; Zhao J.; Szegedi A.; Panagides J. | 2009 | Long-term safety and maintenance of effect of asenapine in patients with acute exacerbation of schizophrenia | No full-text |
| Moon, YE; Jung, HD; Ryu, KH; Moon, DE | 2010 | Effect of 1 mg of prophylactic haloperidol for postoperative nausea and vomiting after gynecological laparoscopic surgery using IV patient controlled analgesia | Not in English |
| Murray, TJ; Kelly, P; Campbell, L; Stefanik, K | 1977 | Haloperiodl in the treatment of stuttering | Pediatric population |
| N/A | 2006 | Delirium treatment at the surgical ward (DELTa S) Treatment of delirium: rivastigmine or haloperidol as primary treatment for delirium in elderly patients with a fractured hip A randomized placebo-controlled study | No full-text |
| NCT00249119, | 2005 | A Study of the Effectiveness and Safety of Risperidone Compared With Haloperidol in Patients With Chronic Schizophrenia | No full-text |
| NCT00249132, | 2005 | A Study of the Effectiveness and Safety of Risperidone Compared With Haloperidol and Placebo in Patients With Chronic Schizophrenia | No full-text |
| NCT00253162, | 2005 | A Study of the Effectiveness and Safety of Risperidone Compared With Placebo in the Treatment of Manic Episodes Associated With Bipolar I Disorder, and the Maintenance of Anti-manic Effectiveness of Risperidone Compared With Haloperidol | No full-text |
| NCT01639599, | 2012 | Optimal Haloperidol Dose for Postoperative Nausea and Vomiting Prevention in High-risk Patients | Duplicate |
| NCT01785290, | 2013 | pRophylactic halopEriDol Use for Delirium in iCu patiEnts With a High Risk for Delirium | Not an RCT |
| NCT02057549, | 2014 | Haloperidol vs Conventional Therapy for Gastroparesis | Duplicate |
| NCT03628391, | 2018 | Haloperidol for Delirium in Adult Critically Ill Patients | No full-text |
| Nordic Dyskinesia Study Group | 1986 | Effect of different neuroleptics in tardive dyskinesia and parkinsonism. A video-controlled multicenter study with chlorprothixene, perphenazine, haloperidol and haloperidol + biperiden. Nordic Dyskinesia Study Group | No placebo arm |
| NTR3207, | 2011 | Haloperidol prophylaxis in older emergency department patients | No full-text |
| NTR533, | 2005 | Delirium treatment at the geriatric ward (DELTa G) Treatment of delirium: rivastigmine or haloperidol as primary treatment for delirium in delirious patients on a geriatric ward. A randomized placebo-controlled study | No full-text |
| NTR6725, | 2017 | Randomised trial of haloperidol for delirium in critically ill patients | No full-text |
| Ortega Soto, HA; Hernandez Avila, CA; Jasso, A; Hasfura Buenaga, CA | 1993 | Carbamazepine vs haloperidol in treatment of manic episodes: a controlled clinical trial | No full-text |
| Page, VJ; Ely, EW; Gates, S; Zhao, XB; Alce, T; Shintani, A; Jackson, J; Perkins, GD; McAuley, DF | 2013 | Effect of intravenous haloperidol on the duration of delirium and coma in critically ill patients (Hope-ICU): a randomised, double-blind, placebo-controlled trial | Duplicate |
| Page, VJ; Ely, EW; Gates, S; Zhao, XB; Alce, T; Shintani, A; Perkins, G; Jackson, JC; McAuley, DF | 2013 | A randomised, double-blind, placebo-controlled trial to compare the early administration of intravenous haloperidol versus placebo in the prevention and treatment of delirium in critically ill ventilated patients (hope-ICU) | Duplicate |
| Perez, C | 1989 | Haloperidol decanoate: results of a 6-month open-label trial in paranoid schizophrenia | No full-text |
| Perry, PJ; Lund, BC; Sanger, T; Beasley, C | 2001 | Olanzapine plasma concentrations and clinical response: acute phase results of the North American Olanzapine Trial | Not an RCT |
| Plath P.; Caspers H.P. | 1975 | Effective treatment of stuttering with haloperidol | Not in English |
| Potkin, SG; Zborowski, J; Wu, JC; Mack, RJ; Sebree, TB; Wallin, BA | 1996 | Brain Imaging to Determine the Effects of Sertindole in Schizophrenic Patients | No full-text |
| Price, WA | 1987 | Antipsychotic effects of verapamil in schizophrenia | No full-text |
| Ramey, T; Giller, EL; English, P; Riesenberg, R; Trivedi, JK; Reddy, JY; Aleksandrovsky, YA; Tochilov, VA; Koe, BK | 2004 | 12-week, double-blind, placebo-controlled study of ziprasidone vs haloperidol for efficacy and maintained treatment effect in acute bipolar mania | Duplicate |
| Rammsayer, TH | 1999 | Neuropharmacological evidence for different timing mechanisms in humans | Healthy volunteers |
| Rammsayer, TH | 1993 | On dopaminergic modulation of temporal information processing | Healthy volunteers |
| Remington, G; Sloman, L; Konstantareas, M; Parker, K; Gow, R | 2001 | Clomipramine versus haloperidol in the treatment of autistic disorder: a double-blind, placebo-controlled, crossover study | Pediatric population |
| Reschke, RW | 1974 | Parenteral haloperidol for rapid control of severe, disruptive symptoms of acute schizophrenia | No full-text |
| Riegger, H; Hollinger, A; Seifert, B; Toft, K; Blum, A; Zehnder, T; Siegemund, M | 2018 | Baden Prevention and Reduction of Incidence of Postoperative Delirium Trial (PRIDe): a phase IV multicenter, randomized, placebo-controlled, double-blind clinical trial of ketamine versus haloperidol for prevention of postoperative delirium | Duplicate |
| Roldan, CJ; Chambers, KA; Paniagua, L; Patel, S; Cardenas-Turanzas, M; Chathampally, Y | 2017 | Randomized Controlled Double-blind Trial Comparing Haloperidol Combined With Conventional Therapy to Conventional Therapy Alone in Patients With Symptomatic Gastroparesis | Duplicate |
| Ruiz-Dangu, DG; Tamayo-Illescas, AJ; Vargas-Ayala, G; Rodriguez-Lopez, L; Jimenez-Saab, NG | 2017 | Efficacy of prophylaxis with haloperidol vs placebo in the prevention of delirium in patients with risk of suffering it hospitalized in an Internal Medicine service | Not in English |
| Ruskin, PE; Nyman, G | 1991 | Discontinuation of neuroleptic medication in older, outpatient schizophrenics. A placebo-controlled, double-blind trial | Not an RCT |
| San Miguel Zamora, MT; Vila Clerigues, MN; Diaz Carrasco, MS; Azorin Sanchez, MD; Fernandez Gomez, V; Robles Garcia, S | 1995 | Extrapyramidal incidence effects in patients with schizophrenia treated with haloperidol either alone or associated with biperidene | No full-text |
| Sanduende, Y; Rama-Maceiras, P; Bautista, A P; Vilela, M; Sarmiento, A; Salamanca, E | 2007 | Haloperidol or droperidol with dexamethasone for antiemetic prophylaxis in laparoscopic cholecystectomy | Not in English |
| Selman, FB; McClure, RF; Helwig, DH | 1976 | Loxapine succinate: a double-blind comparison with haloperidol and placebo in acute schizophrenics | No full-text |
| Selman, FB; McClure, RF; Helwig, H | 1979 | Loxapine succinate: a double-blind comparison with haloperidol and placebo in acute schizophrenics | No full-text |
| Shrivastava, A; Gopa, S | 2000 | Comparative study of risperidone and haloperidol on clinical and psychosocial parameters in treatment of schizophrenia: a randomised open trial | No placebo arm |
| Simpson, GM; Lindenmayer, JP | 1997 | Extrapyramidal symptoms in patients treated with risperidone | Not an RCT |
| Sloman, L; Remington, G; Konstantareas, M; Parker, K | 1998 | Haloperidol versus clomipramine in autistic disorder | No full-text |
| Smit, L; Slooter, A; Devlin, J; Trogrlic, Z; Hunfeld, N; Osse, RJ; Ponssen, H; Brouwers, A; Schoonderbeek, J; Simons, K; et al. | 2021 | Efficacy of halopeRIdol to decrease the burden of Delirium in adult Critically ill patiEnts (EuRIDICE): a Randomized Clinical Trial | No full-text |
| Smit, L; Trogrlic, Z; Devlin, JW; Osse, RJ; Ponssen, HH; Slooter, AJC; Hunfeld, NGM; Rietdijk, WJR; Gommers, D; van der Jagt, M | 2020 | Efficacy of halopeRIdol to decrease the burden of Delirium in adult Critically ill patiEnts (EuRIDICE): study protocol for a prospective randomised multi-centre double-blind placebo-controlled clinical trial in the Netherlands | Not an RCT |
| Soloff, PH; George, A; Nathan, S; Schulz, PM; Ulrich, RF; Perel, JM | 1986 | Amitriptyline and haloperidol in unstable and schizotypal borderline disorders | No full-text |
| Ter Haar H.W. | 1977 | A comparison of chlormethiazole and haloperidol in the treatment of elderly patients with confusion of organic and psychogenic origin: a double-blind crossover study | No full-text |
| Ter Haar H.W. | 1977 | The relief of restlessness in the elderly | Not an RCT |
| Thompson, PM; Bartzokis, G; Hayashi, KM; Klunder, AD; Lu, PH; Edwards, N; Hong, MS; Yu, M; Geaga, JA; Toga, AW; et al. | 2009 | Time-lapse mapping of cortical changes in schizophrenia with different treatments | No placebo arm |
| Tobar E.; Galleguillos T.; Delgado C.; Cornejo R.; Leslie A.; Castillo C.; Galvez L.; Llanos O.; Perez G.; Rojas V. | 2009 | Hypoactive delirium in septic mechanically ventilated patient. Preliminary data of cognitive and psychiatric follow up | No full-text |
| Tollefson, GD; Sanger, T; Beasley, CM | 1996 | The Course of Primary and Secondary Negative Symptoms in a Controlled Trial with Olanzapine | No full-text |
| Tollefson, GD; Sanger, TM | 1997 | Negative symptoms: a path analytic approach to a double-blind, placebo- and haloperidol-controlled clinical trial with olanzapine | Duplicate |
| Tollefson, GD; Sanger, TM; Beasley, CM; Tran, PV | 1998 | A double-blind, controlled comparison of the novel antipsychotic olanzapine versus haloperidol or placebo on anxious and depressive symptoms accompanying schizophrenia | Duplicate |
| Travale, I; Drake, K | 2016 | Haloperidol prophylaxis in cardiac surgery for patients at risk of delirium: a randomized placebocontrolled pilot study | No full-text |
| Travale, IL; Drake, KA; Power, P; Schoenberg, J; Toito, F; Lamy, A | 2016 | Haloperidol prophylaxis in cardiac surgery for patients at risk for delirium: a randomized placebocontrolled pilot study | No full-text |
| Tyrer, P; Oliver-Africano, PC; Ahmed, Z; Bouras, N; Cooray, S; Deb, S; Murphy, D; Hare, M; Meade, M; Reece, B; et al. | 2008 | Risperidone, haloperidol, and placebo in the treatment of aggressive challenging behaviour in patients with intellectual disability: a randomised controlled trial | Duplicate |
| UMIN000001039, | 2008 | A randomized placebo-controlled study of small dose and short duration Haloperidol prophylaxis for elderly at risk for postoperative delirium | No full-text |
| van den Boogaard, M; Slooter, AJC; BrÃ¼ggemann, RJM; Schoonhoven, L; Beishuizen, A; Vermeijden, JW; Pretorius, D; de Koning, J; Simons, KS; Dennesen, PJW; et al. | 2018 | Effect of Haloperidol on Survival Among Critically Ill Adults With a High Risk of Delirium: the REDUCE Randomized Clinical Trial | Duplicate |
| Van Kammen D.P.; Agren H.; Yao J.K.; O'Connor D.T.; Gurklis J.; Peters J.L. | 1994 | Noradrenergic activity and prediction of psychotic relapse following haloperidol withdrawal in schizophrenia | Not an RCT |
| van Keulen, K; Knol, W; Schrijver, EJM; van Marum, RJ; van Strien, AM; Nanayakkara, PWB | 2018 | Prophylactic Use of Haloperidol and Changes in Glucose Levels in Hospitalized Older Patients | Not an RCT |
| Van Schijndel A.; Fransen E.; Van Den Boogaard M.; Van Der Voort P. | 2018 | Haloperidol serum concentrations in critically ill patients included in the REDUCE study | Not an RCT |
| Vasavan Nair, NP; Suranyi-Cadotte, B; Schwartz, G | 1986 | A clinical trial comparing intramuscular haloperidol decanoate and oral haloperidol in chronic schizophrenic patients: efficacy, safety, and dosage equivalence | No placebo arm |
| Veser, F; Zealburg, J; Veser, B; Zhu, Y; Gharabawi, G | 2002 | Oral risperidone in the management of agitated behavior in emergency settings | No full-text |
| Weiner, MF; Tractenberg, RE; Sano, M; Logsdon, R; Teri, L; Galasko, D; Gamst, A; Thomas, R; Thal, LJ | 2002 | No long-term effect of behavioral treatment on psychotropic drug use for agitation in Alzheimer's disease patients | Not an RCT |
| Wright, P; Birkett, M; David, SR; Meehan, K; Ferchland, I; Alaka, KJ; Saunders, JC; Krueger, J; Bradley, P; San, L; et al. | 2001 | Double-blind, placebo-controlled comparison of intramuscular olanzapine and intramuscular haloperidol in the treatment of acute agitation in schizophrenia | Duplicate |
| Wright, P; Birkett, M; Ferchland, I; David, S; Alaka, K; Pullen, P; Brook, S; Reinstein, M; Breier, A | 2000 | A double-blind study of intramuscular olanzapine, haloperidol and placebo in acutely agitated schizophrenic patients | No full-text |
| Wright, P; Birkett, MA; Meehan, K; David, SR; Brook, S; Breier, | 2001 | A double-blind dose response study comparing intramuscular olanzapine, haloperidol and placebo in acutely agitated schizophrenic patients | Duplicate |
| Wright, P; Meehan, K; Birkett, M; Lindborg, SR; Taylor, CC; Morris, P; Breier, A | 2003 | A comparison of the efficacy and safety of olanzapine versus haloperidol during transition from intramuscular to oral therapy | Not an RCT |
| Yocca, F; Marcus, R; Oren, D; Manos, G; Carson, WH; Iwamoto, T; Stock, E | 2005 | Intramuscular Aripiprazole in Acute Schizophrenia: a Pivotal Phase-Three Study | No full-text |
| Zack, M; Lobo, D; Biback, C; Fang, T; Smart, K; Tatone, D; Kalia, A; Digiacomo, D; Kennedy, JL | 2019 | Impulsivity moderates the effects of dopamine D2 and mixed D1Ã¢â‚¬â€œD2 antagonists in individuals with gambling disorder | No placebo arm |
| Zhang, XY; Zhou, DF; Zhang, PY; Shen, YC | 1998 | Effects of risperidone and haloperidol on paroxetine-stimulated neuroendocrine responses and blood sod in schizophrenia | No full-text |
| Zhang, Y; Wang, Q; Reynolds, GP; Yue, W; Deng, W; Yan, H; Tan, L; Wang, C; Yang, G; Lu, T; et al. | 2020 | Metabolic Effects of 7 Antipsychotics on Patients With Schizophrenia: a Short-Term, Randomized, Open-Label, Multicenter, Pharmacologic Trial | No placebo arm |
| Ziemba, C; Foster, G; Neufeld, R; Breuer, B | 1997 | Haloperidol holiday: is it a beneficial vacation for some nursing home residents? | Healthy volunteers |
| Zimbroff D.L.; Kane J.M.; Tamminga C.A.; Daniel D.G.; Mack R.J.; Wozniak P.J.; Sebree T.B.; Wallin B.A.; Kashkin K.B. | 1997 | Controlled, dose-response study of sertindole and haloperidol in the treatment of schizophrenia | Duplicate |
| Zissis, NP; Psaras, M; Lyketsos, G | 1982 | Haloperidol decanoate, a new long-acting antipsychotic, in chronic schizophrenics: double-blind comparison with placebo | No full-text |
