# Supplementary material for: Major adverse cardiac events with haloperidol: A meta-analysis
Source: PLoS One. 2025 Jun 25;20(6):e0326804. doi: 10.1371/journal.pone.0326804 (PMC12194150; doi:10.1371/journal.pone.0326804)
Supplement: S1 Fig — (DOCX) [file pone.0326804.s007.docx]

**S1 Fig 1:** Search Strategy – Medline May 2023 & August 2024

1. exp Haloperidol/

2. haloperidol.tw,kf.

3. haldol.tw,kf.

4. serenace.tw,kf.

5. peridol.tw,kf.

6. or/1-5

7. "randomized controlled trial*".pt.

8. (random* or placebo* or single blind* or double blind* or triple blind*).ti,ab.

9. or/7,8

10. (animals not humans).sh.

11. ((comment or editorial or meta-analysis or practice-guideline or review or letter) not "randomized controlled trial").pt.

12. (random sampl$ or random digit$ or random effect$ or random survey or random regression).ti,ab. not "randomized controlled trial".pt.

13. 9 not (10 or 11 or 12)

14. 6 and 13
