# Supplementary figures and images for: Major adverse cardiac events with haloperidol: A meta-analysis
Source: PLoS One. 2025 Jun 25;20(6):e0326804. doi: 10.1371/journal.pone.0326804 (PMC12194150; doi:10.1371/journal.pone.0326804)

**S1 Fig 2: PRISMA flowchart from June 2023 to August 2024 search update**


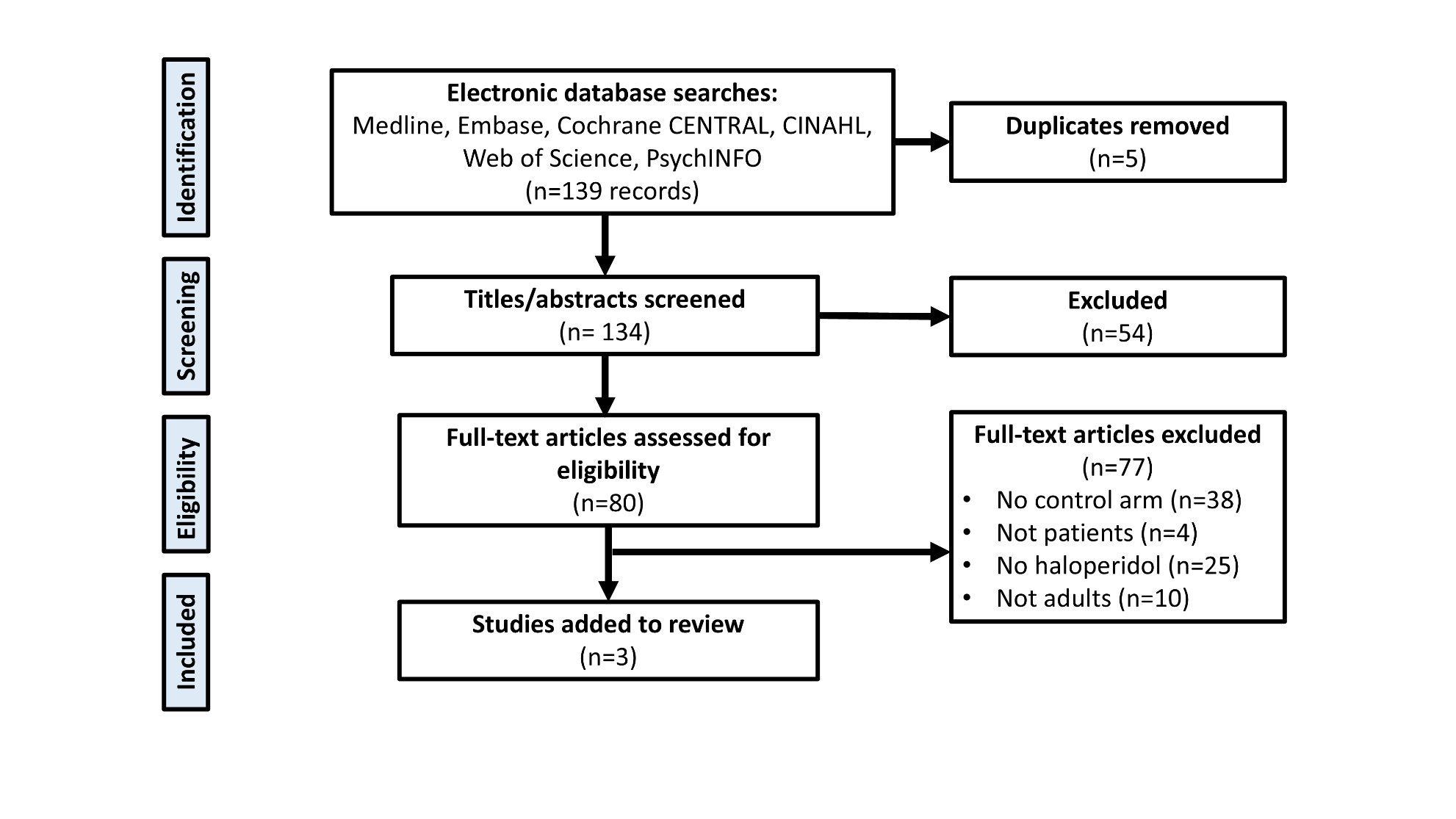

Supplement: S2 Fig — (DOCX) [file pone.0326804.s008.docx]

**S1 Fig 3:** Funnel plot for MACE (left), mortality (right)


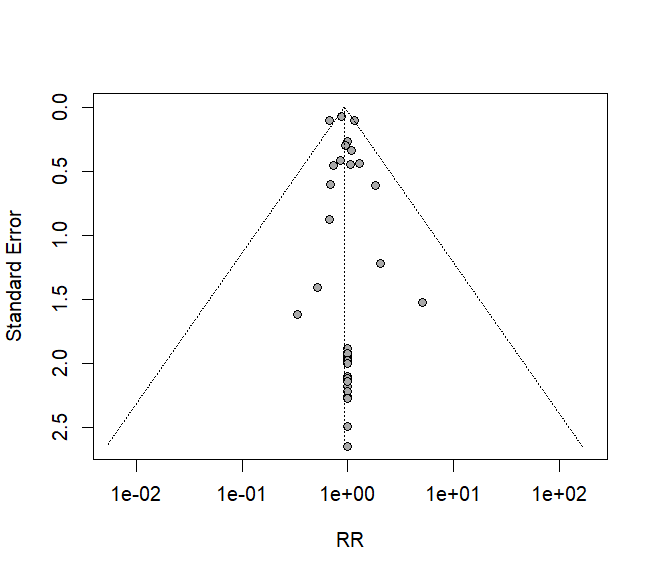

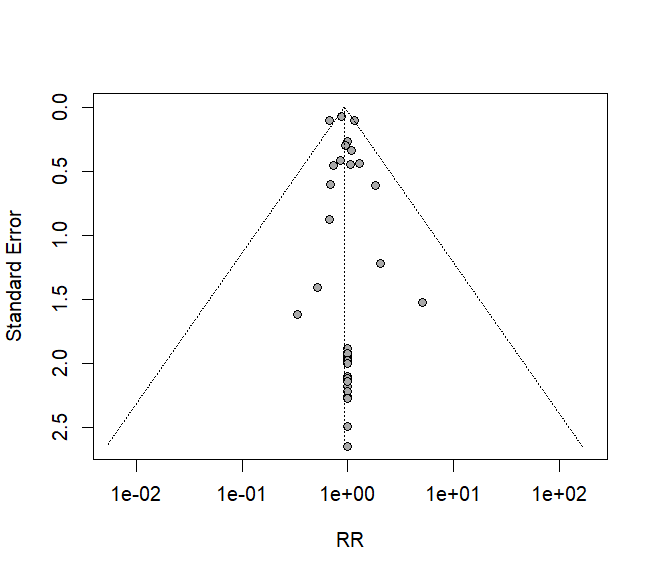

Supplement: S3 Fig — (DOCX) [file pone.0326804.s009.docx]

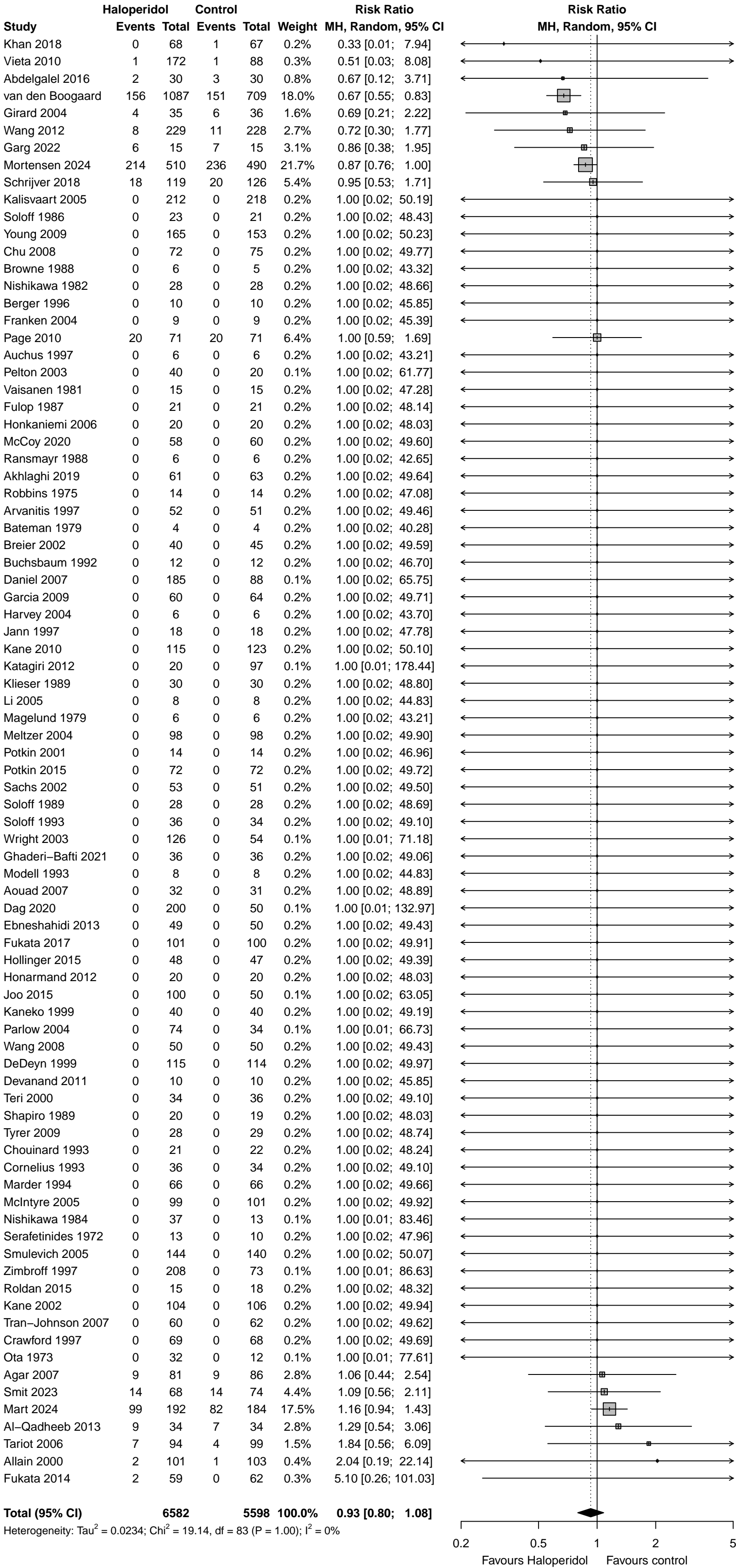

Supplement: S4 Fig — (PDF) [file pone.0326804.s010.pdf]
